# Molecular characterization of a novel serine proteinase from *Trichinella spiralis* and its participation in larval invasion of gut epithelium

**DOI:** 10.1371/journal.pntd.0011629

**Published:** 2023-09-11

**Authors:** Yan Yan Song, Xin Zhuo Zhang, Bo Ning Wang, Min Min Weng, Zhao Yu Zhang, Xin Guo, Xi Zhang, Zhong Quan Wang, Jing Cui

**Affiliations:** Department of Parasitology, Medical College, Zhengzhou University, Zhengzhou, PR China; Seoul National University College of Medicine, REPUBLIC OF KOREA

## Abstract

**Background:**

A novel serine proteinase of *Trichinells spiralis* (TsSPc) has been identified in the excretion/secretion (ES) antigens, but its role in larval invasion is unclear. The aim of this study was to clone and express TsSPc, identify its biological and biochemical characteristics, and investigate its role on larval invasion of gut epithelium during *T*. *spiralis* infection.

**Methodology/Principal findings:**

TsSPc has a functional domain of serine proteinase, and its tertiary structure consists of three amino acid residues (His88, Asp139 and Ser229) forming a pocket like functional domain. Recombinant TsSPc (rTsSPc) was expressed and purified. The rTsSPc has good immunogenicity. On Western blot analysis, rTsSPc was recognized by infection serum and anti-rTsSPc serum, natural TsSPc in crude and ES antigens was identified by anti-rTsSPc serum. The results of qPCR, Western blot and indirect immunofluorescence test (IIFT) showed that TsSPc was expressed at diverse stage worms, and mainly localized at cuticle, stichosome and intrauterine embryos of this nematode. The rTsSPc had enzymatic activity of native serine protease, which hydrolyzed the substrate BAEE, casein and collagen I. After site directed mutation of enzymatic active sites of TsSPc, its antigenicity did not change but the enzyme activity was fully lost. rTsSPc specifically bound to intestinal epithelium cells (IECs) and the binding sites were mainly localized in cell membrane and cytoplasm. rTsSPc accelerated larval invasion of IECs, whereas anti-rTsSPc antibodies and TsSPc-specific dsRNA obviously hindered larval invasion.

**Conclusions:**

TsSPc was a surface and secretory proteinase of the parasite, participated in larval invasion of gut epithelium, and may be considered as a candidate vaccine target molecule against *Trichinella* intrusion and infection.

## Author summary

A novel *Trichinella spiralis* serine proteinase (TsSPc) has been identified from ES antigens of *T*. *spiralis* intestinal infective larvae (IIL) by immunoproteomics. The objective of this study was to investigate the biological characteristics of TsSPc and its role in larval invasion. rTsSPc was cloned and expressed, and rTsSPc and native TsSPc in crude and ES antigens of various worm stages were recognized by anti-rTsSPc antibodies, suggesting that TsSPc is a secretory protein with good antigenicity. Results of qPCR, Western blot and indirect immunofluorescence test (IIFT) showed that TsSPc was expressed at diverse stages, and mainly localized at cuticle, stichosome and intrauterine embryos of this nematode. rTsSPc had enzymatic activity of natural serine proteinases for hydrolyzing the substrates (BAEE, casein and collagen I), but enzymatic activity of rTsSPc was completely lost after its enzyme active sites were mutated. IIFT, Far western and confocal microscopy showed that rTsSPc specifically bound to intestinal epithelial cells (IECs), and the binding site was principally localized in cell membrane and cytoplasm. An *in vitro* larval invasion test revealed that rTsSPc evidently accelerated larval invasion of IECs; whereas anti-rTsSPc antibodies and TsSPc-specific dsRNA significantly impeded larval invasion. The results indicated that TsSPc participated in larval invasion of host gut mucosa, and may be considered as a candidate vaccine target for intercepting *Trichinella* intrusion and infection.

## Introduction

Trichinellosis is an important zoonotic parasitic disease caused by the consumption of raw or undercooked animal meat infected with *Trichinella* sp. muscle larvae [1]. From 2009 to 2020, eight outbreaks of human trichinellosis caused by infected pork or pork products were reported in China [2]. *Trichinella* infection not only has a threat to public health, but also possesses a risk to animal food safety and global meat trade [3]. Therefore, it is necessary to develop preventive vaccines to block the transmission of *Trichinella* infection in domestic pigs and eliminate the infective larvae in animals consumed as food [4,5].

After being ingested, the encapsulated *Trichinella spiralis* muscle larvae (ML) are released from the capsules with the aid of digestive fluid and activated into intestinal infectious larvae (IIL) by exposure to gut contents or bile. The IIL penetrate intestinal epithelial cells (IECs) and establish their multicellular niche, where they molt for the first time 10 hours post infection (hpi), and then molt three times to adult worm (AW) stage 31 hours post infection (hpi) [6]. After copulation, pregnant adult females produce the next generation of newborn larvae (NBL), which enter the bloodstream, penetrate the skeletal muscles, and develop into the encapsulated ML and complete their life cycle [7]. The IIL penetration of IECs is the essential step for *T*. *spiralis* infection and pathogenesis. Gut epithelium is the first natural physical barrier against the invasion of the intestinal parasites, and also the main site of the interaction between parasite and host [8,9], but the mechanism of the IIL invading IECs has not been fully clarified [10,11]. The identification and characterization of larval invasion-related molecules will be favorable for elucidating the mechanism of *T*. *spiralis* invasion and developing preventive vaccines to prevent *T*. *spiralis* invasion [12,13].

Excretion/secretion (ES) antigens of *T*. *spiralis* IIL contain many kinds of proteases; they are first exposed to the host intestinal mucosa and play an important role in disrupting the host intestinal epithelial barrier [14]. By using proteomics/immune proteomics analysis, various serine proteases were identified in *T*. *spiralis* IIL ES products [15,16], and they might participate in larval invasion of the host gut epithelium [17,18]. Therefore, serine proteases might be the main candidate target molecules for the vaccines against *Trichinella* invasion of gut mucosa [19,20]. In previous studies, a novel serine proteinase from *T*. *spiralis* (TsSPc, Genbank: U62659.1) was identified in the ES and surface proteins of the IIL stage [21–23]. The serine proteinase was also present in AW and NBL stages of *T*. *spiralis* [24]. But, the biological characteristic of TsSPc and its function in *T*. *spiralis* life cycle has not been reported in the literatures so far.

The aim of this study was to clone and express TsSPc, identify its biological and biochemical characteristics, and investigate its action on larval invasion of gut epithelium during *T*. *spiralis* infection.

## Materials and methods

### Ethics statement

This study was performed in the light of National Guidelines for Experimental Animal Welfare (Minister of Science and Technology, People’s Republic of China, 2006). All animal experiments in this study were approved by the Life Science Ethics Committee of Zhengzhou University (No. ZZUIRB GZR 2021–0044).

### *Trichinella*, cells and experiment animals

The species of *Trichinella* spp. used in this experiment was *T*. *spiralis* isolate (ISS534) obtained from an infected domestic pig in Nanyang, Henan Province of China [19]. Human colon epithelial cell line Caco-2 and mouse striated muscle myoblast C2C12 were obtained from the Cell Resource Center of the Shanghai Institute for Biological Sciences of the Chinese Academy of Sciences. Female BALB/c mice aged 4–6 weeks were purchased from Henan Experimental Animal Center, China.

### Worm collection and antigen preparation

Murine carcasses infected with *T*. *spiralis* at 35 dpi were digested by using artificial digestion fluid (1% pepsin, 0.75% HCL and 0.9% NaCl) at 37 °C for 3 h to collect the ML [24]. The IIL and AW were recovered from small intestine at 6 hpi, 3 and 6 dpi, respectively [25,26]. Adult females were cultivated in RPMI-1640 supplemented with 10% fetal bovine serum (FBS; Gibco, USA) for 24 h at 37 °C and 5% CO_2_, and the NBL were collected as described previously [27,28]. The soluble crude antigens of diverse *T*. *spiralis* stage worms (ML, IIL, AW and NBL) and their ES antigens were prepared as reported before [29,30].

### Cloning and expression of rTsSPc

The full-length cDNA sequence of TsSPc gene was retrieved from GenBank (GenBank: U62659.1), and its physicochemical properties and structures were analyzed and predicted using the online website for ExPASy (https://www.expasy.org/) [31].

Total RNA was extracted from the 6 h IIL using TRIzol (Invitrogen, USA). TsSPc-specific primers were designed with restriction sites of *BamH*I and *Sal*I (bold) (5′-AA**GGATCC**ATTGTAGGCGGTAGTGAT-3′, 5′-GC**GTCGAC**TTAGGAAGAGTAC AATT-3′). The complete TsSPc cDNA sequence was amplified by PCR. The PCR product was cloned into the pQE-80L carrying a His-tag at N-terminus (Novagen, USA), and recombinant pQE-80L/TsSPc was transformed into *E*. *coli* BL21 (Novagen). The expression of rTsSPc was induced with 0.5 mM isopropyl β-D-1-thiogalactopyranoside (IPTG) at 37 °C for 6 h [10]. A Ni-NTA-Sefinose resin containing His tag (Sangon Biotech, Shanghai, China) was used to purify rTsSPc [32]. The expression of rTsSPc protein was analyzed by SDS-PAGE and Western blotting.

### Preparation of anti-rTsSPc serum

Ten mice were subcutaneously immunized with 20 μg rTsSPc mixed with complete Freund’s adjuvant. Boost immunization was given two times with 20 μg rTsSPc mixed with incomplete Freund’s adjuvant at an interval of two weeks [33]. At two weeks after the third immunization, the tail blood of mice was collected to isolate anti-rTsSPc immune serum. Anti-rTsSPc IgG titer of all immunized mice was determined by conventional indirect ELISA as reported before [34]. In brief, ELISA plates were coated with 1.5 μg/ml rTsSPc at 4 °C overnight. After washing with PBS (pH 7.4) containing 0.05% Tween-20 (PBST), the plate was blocked with 5% skim milk at 37 °C for 2 h. Following washes, serially diluted anti-rTsSPc immune serum was added and incubated at 37°C for 2 h, then incubated at 37 °C for 1 h with HRP-conjugated goat anti-mouse IgG (1:10000; Southern Biotech, USA). Coloration was developed with OPD (Sigma, USA) plus H_2_O_2_, the reaction was terminated using 2 M H_2_SO_4_. The OD values at 492 nm were assayed by a microplate reader (Tecan, Schweiz, Switzerland) [35].

### Western blotting analysis

Proteins samples consisted of rTsSPc, crude antigens of ML, IIL, 3 d AW, 6 d AW and NBL, and ES antigens of ML, IIL, 3 d AW and 6 d AW. The proteins were separated on SDS-PAGE with 12% separation gel, subsequently transferred onto the membranes (Millipore, USA) at 0.25 A for 90 min in a wet transfer cell (Bio-Rad, USA) [34,36]. The membrane was blocked by 5% skim milk in Tris–buffered saline with 0.05% Tween-20 (TBST) at room temperature for 2 h, and incubated with 1:100 dilutions of different sera (anti-rTsSPc serum, *T*. *spiralis*-infected mouse serum collected at 35 dpi, and normal mouse pre-immune serum) at 4 °C overnight. Following being washed, the membrane was incubated with 1:10 000 dilutions of HRP-conjugated goat anti-mouse IgG (Southern Biotechnology, USA) at 37 °C for 1 h. The membrane was colored by use of 3,3’-diaminobenzidine tetrahydrochloride (DAB; Sigma), and stopped by washing the membrane with deionized water [37–39]. Color development of the membrane was also performed by the enhanced chemiluminescence (ECL) kit (CWBIO, Beijing, China) [32], and the relative expression level of the TsSPc protein at various *T*. *spiralis* phases was determined with ImageJ software.

### Real-time quantitative PCR (qPCR) assay

Total RNA from various *T*. *spiralis* worm stages (ML, IIL, 3 d and 6 d AW, and NBL) was isolated using TRIzol reagent (Invitrogen). The TsSPc mRNA transcription level at diverse worm stages was ascertained by qPCR as described previously [40,41]. TsSPc-specific primers for qPCR were 5′-TTGTTTTCAGCTGCTTGCGG-3′ and 5′- ATGGGGAACGGCATCACTAC-3′. The relative TsSPc mRNA expression level was normalized by subtracting the mRNA expression level of the *T*. *spiralis* housekeeping gene GAPDH (GenBank: AF452239) [38] and then calculated on the basis of the comparative Ct (2^−ΔΔCt^) method [42]. Each experiment had three replicates.

### Indirect immunofluorescence test (IIFT)

To investigate expression and worm localization of natural TsSPc in different stages of *T*. *spiralis*, IIFT was performed as reported previously [43,44]. Whole intact worms of various *T*. *spiralis* stages (ML, IIL, AW and NBL) were fixed with 4% paraformaldehyde and embedded in paraffin, and 2-μm-thick worm cross-sections were cut with a microtome [45]. Whole worms and worm cross-sections were blocked with 5% goat serum at 37 °C for 1 h, washed three times in PBS, and then incubated with 1:10 dilutions of anti-rTsSPc serum, infection serum and pre-immune serum. Goat anti-mouse IgG-Alexa Fluor 488 conjugate (1:100; Abways, Shanghai, China) was used as the secondary antibody. After washes again, whole worms and cross-sections were examined by fluorescence microscopy (Olympus, Japan) [40,46].

### Assay of rTsSPc enzyme activity by using substrate BAEE

Since rTsSPc is expressed in the form of inclusion bodies, rTsSPc is first refolded through dialysis and renaturation method [47]. To assay the enzymatic activity of rTsSPc, the serially diluted rTsSPc (0, 0.01, 0.02, 0.04, 006, 0.08, 0.10 μg/μl) was first pre-incubated at 37 °C for 15 min in various pH buffer (pH 3.0–5.0 sodium acetate, pH 6.0–7.0 sodium phosphate, pH 8.0–9.0 Tris-HCl, and pH 10.0–10.5 sodium bicarbonate). Subsequently, the substrate N-benzoyl-L-arginine-ethylester (BAEE; BBI, Shanghai, China) was added to the reaction mixture and incubated at different temperatures (20, 30, 37, 40, 50, 60, 70, and 80 °C) for 15 min, and the absorbance at 253 nm was measured using a spectrophotometer [48]. To evaluate the effect of different metal ions on rTsSPc enzyme activity, metal ions (Ca^2+^, Fe^2+^, Zn^2+^, Mg^2+^, Ni^2+^, and Mn^2+^) were added to the reaction system [12,49]. Moreover, different protease inhibitors (1 mM PMSF, 20 μM E-64, 2 mM 1, 10–Phe, 10 μM Pepstatin or 10 mM EDTA) were also used to evaluate the effect of these inhibitors on rTsSPc activity. The denatured rTsSPc which was inactivated at 100 °C for 5 min was used as a positive control for inhibition. Each sample has three replicates.

### Assay of rTsSPc activity using casein as substrate

The rTsSPc proteolysis activity was measured by substrate gel electrophoresis containing 0.1% casein substrate [50,51]. Briefly, the rTsSPc (2 μg/lane) mixed with the loading buffer lacking β-mercaptoethanol was separated by SDS-PAGE [38]. The substrate gel was loaded with the rTsSPc and run at 120 V for approximately 120 min under non-reducing conditions. After electrophoresis, gels were incubated with renature buffer for 40 min to remove the SDS at room temperature and incubated in 50 mM Tris-HCl buffer (pH 8.0) for 48 h at 37 °C. Finally, the gels were stained with Coomassie brilliant blue for 2 h and destained until clear translucent bands appeared against the dark blue background [52]. In addition, heat-inactivated rTsSPc, 1 mM PMSF+rTsSPc and 5 μg recombinant *T*. *spiralis* serine protease inhibitor (rTsSPI)+rTsSPc were used as the negative control.

Moreover, the proteolytic activity of rTsSPc was assayed by using azocasein (Sigma). The effect of pH on rTsSPc enzymatic activity to degrade azocasein was measured at pH 5.5–10.5 [53]. Briefly, 2 μg rTsSPc proteins were mixed with 25 μl buffer and 20 μl azocasein substrate (10 mg/ml) and incubated for at 37°C 16 h. Then, the assay was stopped using 50 μl 12% trichloroacetic acid at room temperature for 15 min and centrifuged at 12 000 × *g* for 20 min. The collected supernatants were neutralized by equivolumetric NaOH (1 M) and the absorbance (OD value) at 405 nm was measured. All reactions were performed in triplicate [54]. rTsSPc was also pre-incubated with diverse enzyme inhibitors at 37 °C for 30 min, and then incubated with azocasein at 37 °C for 16 h [36]. Inhibitors used in this study included E-64 (20 μ M), PMSF (1 mM), 1,10-phenanthroline (2 mM), and pepstatin (10 μM). Inhibition rate (%) = OD values of rTsSPc with inhibitors/ OD values of rTsSPc without inhibitors ×100%. All assays have three independent replicates.

### rTsSPc active site mutation

In order to further investigate the rTsSPc enzyme activity, the site directed mutagenesis was also performed in this study. Three enzyme active sites at 83 aa (His), 139 aa (Asp) and 229 aa (Ser) of TsSPc were mutated to inactive amino acid (Ala) [55]. Mutant sequence was synthesized by generic Biotech (Suzhou, China) and introduced into *Escherichia coli*. The mutant TsSPc (MTsSPc) was also induced using 0.5 mM IPTG at 37 °C for 6 h and purified with Ni-NTA-Sefinose resin and analyzed on Western blotting [36,37]. Moreover, the enzymatic activity of MTsSPc was determined by using the substrate BAEE.

### Determination of rTsSPc degrading collagen I by SDS-PAGE

Murine collagen I (Sigma) was used to determine the hydrolytic function of rTsSPc. Various doses of rTsSPc (0.5, 1.0, 1.5 and 2.0 μg) were incubated with 2 μg collagen I at different pH (7.0, 8.0 and 9.0) at 37 °C overnight [56,57]. Simultaneously, rTsSPc was first pre-incubated with 1 mM PMSF for 30 min, and then PMSF-pre-incubated rTsSPc, MTsSPc, or trypsin was incubated with 2 μg collagen I overnight at 37 °C. Reactions were ceased by the addition of sample buffer contained 2% SDS and 1% β-mercaptoethanol. Samples were denatured at 100 °C for 5 min and separated on 12% gels [48].

### Far western blotting analysis of rTsSPc binding to Caco-2 cells

The binding of rTsSPc and Caco-2 cells was investigated by Far western blotting analysis as previously described [11]. In brief, Caco-2 cell proteins were first separated by SDS-PAGE, and then transferred to the membrane (Millipore). The membrane was cut into strips and blocked with 5% skimmed milk at 37 °C for 2 h, and then incubated with 20 μg/ml rTsSPc for 2 h at 37 °C. After washing with TBST, strips were probed using anti-rTsSPc immune serum. Following washing using TBST, strips were incubated with HRP-conjugated anti-mouse IgG (1:10000, Sigma) at 37 °C for 1 h. After washing, coloration was developed by DAB (Sigma) [37,58].

### IIFT analysis of rTsSPc binding with Caco-2 cells and cellular localization

IIFT analysis of rTsSPc binding with Caco-2 cells and cellular localization was performed as reported previously [8]. The Caco-2 and C2C12 cells were grown to about 90% confluence on glass coverslips in MEM medium in a culture plate for 36 h. The viable cell monolayer was incubated for 2 h at 37 °C with 20 μg/ml rTsSPc. The IIL ES antigens and PBS were utilized as a positive and negative control, respectively. After washing with PBS, the monolayer was fixed with 4% formaldehyde for 20 min. The monolayer was probed by 1:10 dilutions of anti-rTsSPc serum, infection serum or pre-immune serum, subsequently incubated with 1:100 dilutions of anti-mouse IgG-Alexa Fluor 488 conjugate (Abways) at 37 °C for 1 h. The monolayer cell nuclei were dyed with 4’,6-diamidino-2-phenylindole (DAPI). After being washed again, the cells were observed by fluorescent microscopy (Olympus, Tokyo, Japan) [34]. Furthermore, the cellular localization of rTsSPc within Caco-2 cells was further examined under a laser scanning confocal microscopy [59]. Each experiment was done in triplicate.

### IIFT analysis of rTsSPc binding with normal murine gut epithelium

To determine the ability of rTsSPc binding to normal murine gut epithelium, small intestines from normal mice were fixed with paraformaldehyde, 3-μm intestinal tissue sections were prepared, and IIFT was performed as described previously [35]. After intestinal sections were blocked using 5% goat serum, they were incubated with rTsSPc or IIL ES antigens (20 μg/ml) for 1 h at 37 °C, and the subsequent procedures were the same as those described in the IIFT section. Each experiment was done in triplicate.

### RNA interference (RNAi)

Three pairs of primers of TsSPc-specific dsRNA targeting to the TsSPc domain cDNA sequence was designed (Table 1). *T*. *spiralis* trypsin (TsTryp, GenBank: XM_003381619.1) and *T*. *spiralis* novel dipeptidyl peptidase 1 (TsDPP1, GenBank: XP_003379334.1) were used to confirm TsSPc-dsRNA specificity. Additionally, green fluorescent protein (GFP) dsRNA was also prepared as a negative control [40,60]. Diverse dsRNAs were transfected into *T*. *spiralis* ML by electroporation and cultured in RPMI 1640 medium at 37 °C for 1–3 d. The TsSPc transcription and expression were assessed by qPCR and Western blotting as previously reported [30,36], and the GAPDH was used as the internal control. TsSPc protein expression level was assessed by densitometry measured with Image J software [12].

**Table 1:** TsSPc-specific dsRNA primers with T7 RNA polymerase promoter sequences*.

Primers	Sequences
T7-TsSPc-F1	5′-GATCAC**TAATACGACTCACTATAGGG**AACCCAAATTACCCGGTGGG-3′
T7-TsSPc-R1	5′-GATCAC**TAATACGACTCACTATAGGG**GCAATTTGTCATCCTTTTTCGGC-3′
T7-TsSPc-F2	5′-GATCAC**TAATACGACTCACTATAGGG**TGGAAACGGTGCTGGAAGTT-3′
T7-TsSPc-R2	5′-GATCAC**TAATACGACTCACTATAGGG**TGTCCACATCTACTTGTTTCAGTG-3′
T7-TsSPc-F3	5′-GATCAC**TAATACGACTCACTATAGGG**GAAGCTTCTGCCCGAAAGGT-3′
T7-TsSPc-R3	5′-GATCAC**TAATACGACTCACTATAGGG**AGCCAGGATGTTTACTATCACCA-3′
T7-GFP-F	5′-GATCAC**TAATACGACTCACTATAGGG**TCCTGGTCGAGCTGGACGG-3′
T7-GFP-R	5′-GATCAC**TAATACGACTCACTATAGGG**TTCTCGTTGGGGTCTTTG-3′

* The underlined sequence was the protective bases; the bold bases were the T7 promoter sequence.

### The *in vitro* invasion assay

In order to determine the role of TsSPc in larval invasion of gut epithelium, the *in vitro* invasion assay was performed as described before [61,62]. The Caco-2 cell monolayers were overlaid with 100 IIL suspended in semisolid medium with different doses of rTsSPc (0, 5, 10, 15, 20 and 25 μg/ml), 1:100–1:1600 dilutions of anti-rTsSPc serum, infection serum or pre-immune serum, and incubated in 5% CO_2_ for 2 h at 37 °C [17,63]. The IIL ES antigens and BSA were served as a positive and negative control. Moreover, to ascertain the effect of rTsSPc enzymatic activity on larval invasion, MTsSPc, heat-inactivated rTsSPc, and PMSF+rTsSPc were used in the present study. The suppressive role of TsSPc-dsRNA on larval invasion of Caco-2 cell monolayers was also investigated as reported before [39]. The number of larvae invaded into the cell monolayers was observed and counted under an inverted phase-contrast microscope (Olympus, Japan). The invaded larvae were active and locomotive in the monolayer, whereas the non-invaded larvae were still suspended in medium and spirally coiled [38]. Each invasion assay had three replicates.

### Statistical analysis

All data were analyzed using SPSS 22.0 software. The results are displayed as mean ± standard deviation (SD). One-way ANOVA was used to analyze the transcription and expression levels of TsSPc among different groups. The chi square test was used to analyze the invasion and TsSPc expression of various groups of larvae after RNAi. *P* < 0.05 is considered statistically significant.

## Results

### Bioinformatics analysis of TsSPc

**Fig 1:**
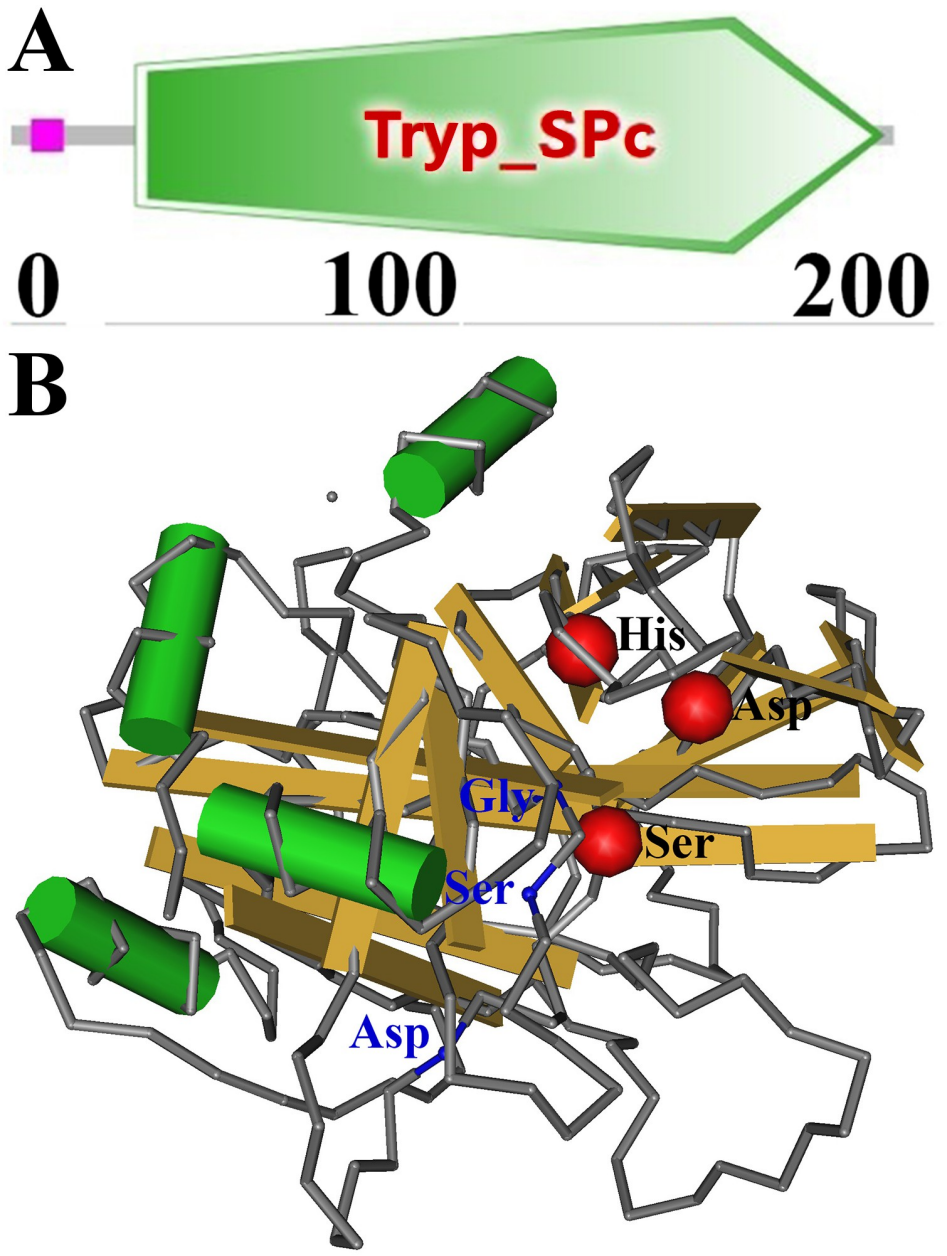
Predicted 3-dimensional structure of TsSPc protein. **A**: Predicted functional domains of TsSPc (Tryp-SPc). **B**: Predicted TsSPc-specific three enzymatic activity sites (His, Asp, and Ser) are highlighted with red color. Three substrate binding sites (Asp, Ser and Gly) are signed as blue.

Complete TsSPc cDNA sequence is 834 bp encoding 277 aa, molecular weight (MW) is 28.33 kDa and pI is 8.69. The TsSPc contains a signal peptide, has obvious hydrophobicity at the N-terminus. Subcellular localization prediction showed that TsSPc is mainly located outside the cell and may be a secretory protein. TsSPc structural prediction showed a serine protease domain (Tryp_SPc) at 38–267 aa (Fig 1A). Three-dimensional structure of TsSPc contains 3 active sites (His, Asp, and Ser) forming a pocket like functional domain (Fig 1B).

### Western blot analysis of the rTsSPc

**Fig 2:**
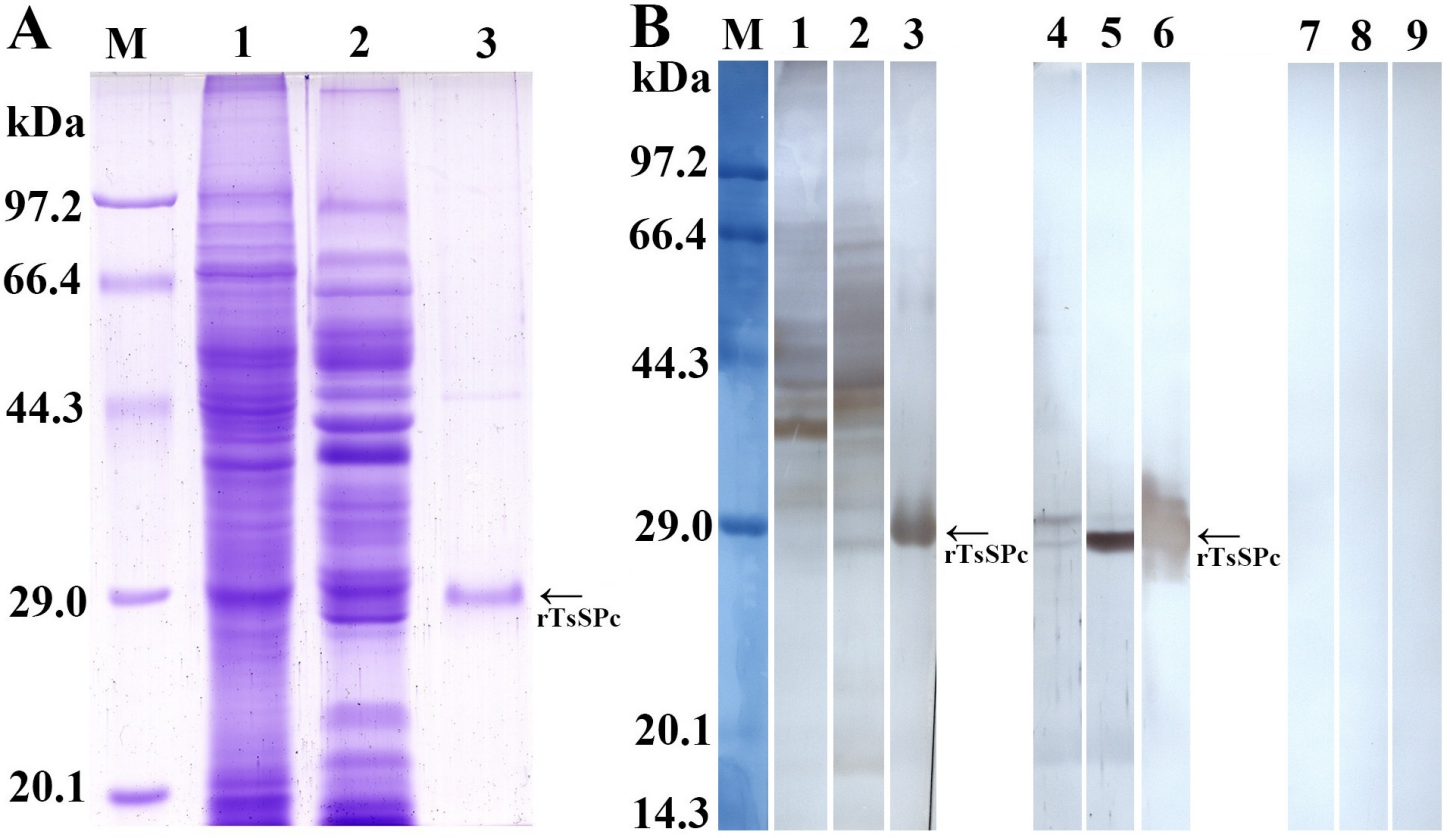
Identification of rTsSPc antigenicity. **A**: SDS-PAGE analysis of rTsSPc. Lane M: protein marker; lane 1: IIL crude antigens; lane 2: IIL ES antigens; lane 3, rTsSPc. **B**: Western blotting of rTsSPc antigenicity. IIL crude antigens (lane 1), IIL ES antigens (lane 2) and rTsSPc (lane 3) were recognized by infection serum; native TsSPc in IIL crude antigens (lane 4), IIL ES antigens (lane 5) and rTsSPc (lane 6) were identified by anti-rTsSPc serum. IIL crude antigens (lane 7), IIL ES antigens (lane 8), and rTsSPc (lane 9) were not recognized by normal mouse serum.

After induction with IPTG, the fusion protein with the His-tag was expressed in *E*. *coli* BL21 harboring pQE-80L/TsSPc. The rTsSPc protein was purified using a Ni–NTA-Sepharose column. SDS–PAGE analysis revealed that rTsSPc had a clearly visible individual band, and its MW (25.18 kDa) was consistent with its predicted size (Fig 2A). Western blot analysis showed that rTsSPc was recognized by anti-rTsSPc serum and infection serum, but not by normal murine serum (Fig 2B). Moreover, anti-rTsSPc serum also recognized natural TsSPc of 28.33 kDa and 25.18 kDa in IIL crude antigens, as well as natural TsSPc of 25.18 kDa in IIL ES antigens, indicating that natural TsSPc is a secretory protein of the nematode.

### Humoral immune response elicited by rTsSPc immunization

**Fig 3:**
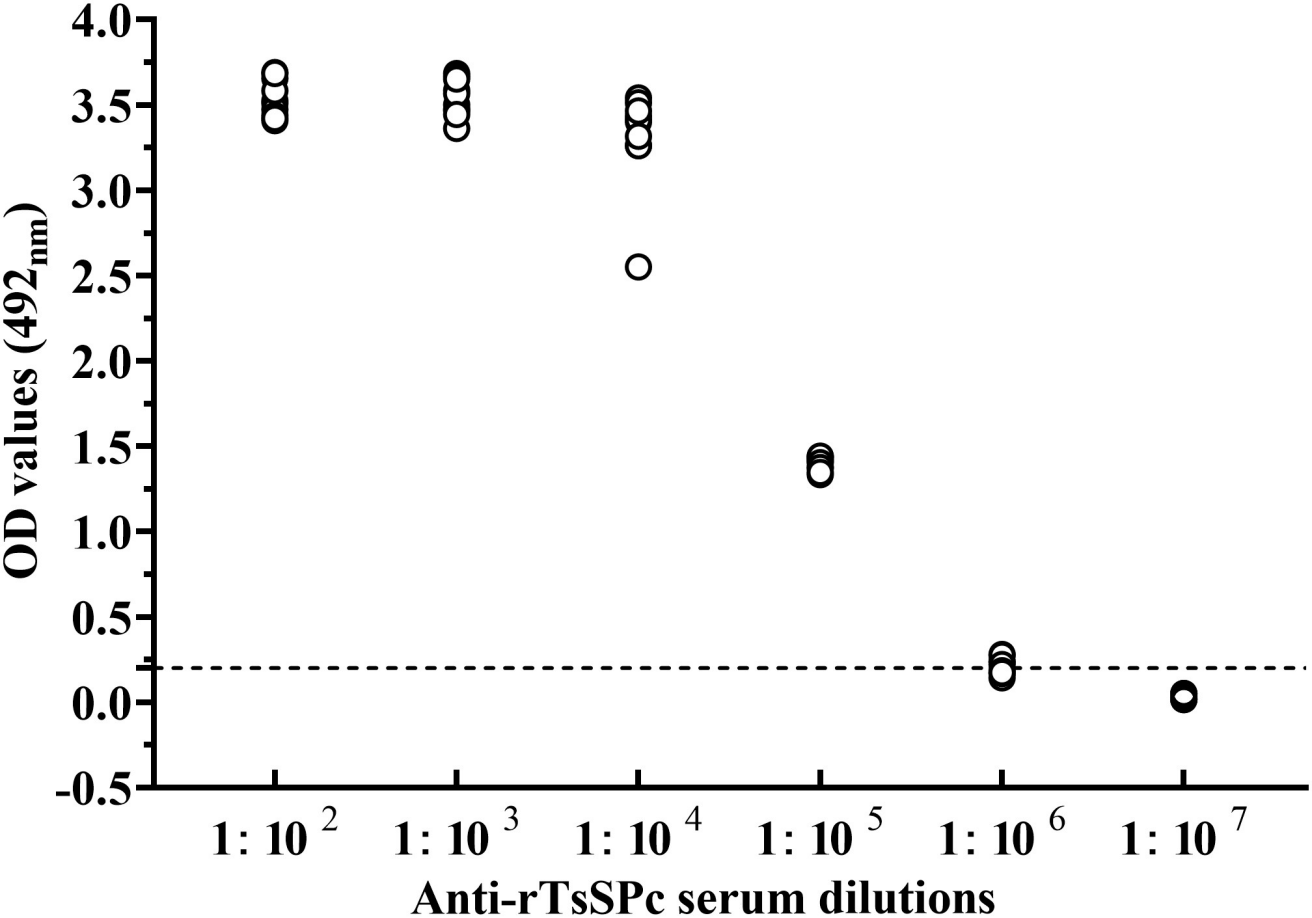
Serum anti-rTsSPc IgG titers measured by ELISA. The plate was coated using 1.5 μg/ml rTsSPc incubated at 4 °C overnight. After blockage with 5% skimmed milk and washes, the plate were probed with various dilutions of ten immune serum samples at 37 °C for 2 h. Normal murine sera (n = 30) diluted at 1:100 were determined as negative controls. HRP-conjugated IgG was used as the second antibodies. The coloration was developed by using the substrate OPD. The absorbance at 492 nm was measured after adding 2 M H_2_SO_4_. The cut-off value (0.201) is represented by a dotted line.

To assess the antibody response elicited by rTsSPc immunization, the titer of anti-rTsSPc IgG at two weeks after the third immunization was measured by ELISA. The results showed that anti-rTsSPc IgG titer reached 1:10^5^ after three immunizations (Fig 3), indicating that rTsSPc has good antigenicity.

### Transcription and expression level of rTsSPc in diverse *T*. *spiralis* stages

**Fig 4:**
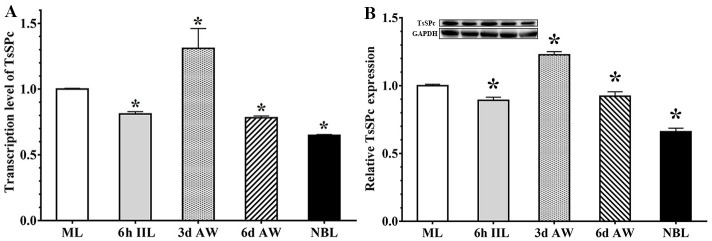
qPCR and Western blot analysis of TsSPc expression at different stages of *T*. *spiralis*. **A**: qPCR assay of TsSPc transcription levels in diverse *T*. *spiralis* phases. Relative transcription level of TsSPc at the 3 d AW stage was evidently higher than those of other worm stages. **B**: Western blot analysis of TsSPc expression levels at diverse *T*. *spiralis* phases. Expression levels of TsSPc protein in crude antigens of diverse *T*. *spiralis* phase (ML, IIL, 3 d AW, 6 d AW and NBL) were ascertained by Western blot with 1:100 dilutions of anti-rTsSPc serum. The graph reveals the relative protein expression assayed with densitometry in 3 independent experiments. * *P* < 0.05 compared to the ML stage.

qPCR analysis showed that the TsSPc gene was transcribed at different stages in *T*. *spiralis* life cycle (ML, IIL, 3 d and 6 d AW, and NBL). Compared to the ML stage, TsSPc had the highest transcription level in 3 d AW stage (*F* = 12.518, *P* < 0.05) (Fig 4A), suggesting that TsSPc plays an important role in intestinal *T*. *spiralis* phase. The results of Western blot analysis demonstrated that native TsSPc of 25.18 kDa in crude antigens of various worm stages were also probed by anti-rTsSPc serum, and the TsSPc expression level in 3 d AW stage was obviously higher than the ML stage (*F* = 209.562, *P* < 0.0001) (Fig 4B). These results further indicated that the TsSPc was transcribed and expressed at various developmental phases.

**Fig 5:**
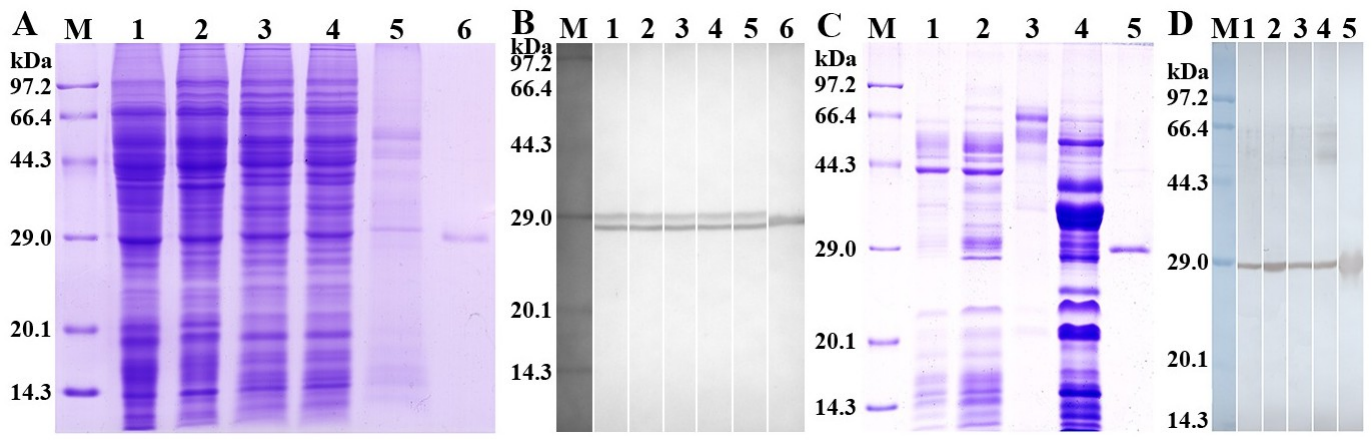
Western blot analysis of TsSPc expression in different stages of *T*. *spiralis*. **A**: SDS-PAGE of soluble crude proteins of diverse worm phases. Lane M: protein marker; Lane 1: ML; lane 2: IIL; lane 3: 3 d AW; lane 4: 6 d AW; lane 5: NBL; Lane 6: rTsSPc. **B**: Western blot analysis of native TsSPc in soluble somatic proteins of ML (Lane 1), IIL (Lane 2), 3 d AW (Lane 3), 6 d AW (Lane 4) and NBL (Lane 5) probed using anti-rTsSPc serum, and rTsSPc (lane 6) recognized by anti-rTsSPc serum was used as positive control. **C**: SDS-APGE analysis of ES antigens of different *T*. *spiralis* stages. Lane M: protein marker; lane 1–4: ES antigens of *T*. *spiralis* ML (lane 1), IIL (lane 2), 3 d AW (lane 3), 6 d AW (lane 4), and rTsSPc (lane 5). **D**: Western blotting of ES antigens of diverse *T*. *spiralis* phases. Native TsSPc in ES antigens of *T*. *spiralis* ML (Lane 1), IIL (Lane 2), 3 d AW (Lane 3), 6 d AW (Lane 4), and rTsSPc (Lane 5) were identified by anti-rTsSPc serum.

To further investigate the expression of TsSPc in worm somatic soluble and ES proteins of the parasite, crude antigens of diverse *T*. *spiralis* stages were isolated on SDS–PAGE analysis (Fig 5A). Western blot results revealed that native TsSPc in crude antigens of diverse phases (ML, IIL, 3 d and 6 d AW and NBL) was identified by anti-rTsSPc serum (Fig 5B). It is interesting that two clear bands (28.33 and 25.18 kDa) in crude antigens were recognized by anti-rTsSPc serum, suggesting that they might be precursor (28.33 kDa) and activated state (25.18 kDa) of TsSPc. Furthermore, the ES antigens from diverse stage worms (ML, IIL, 3d AW, and 6d AW) were also isolated on SDS-PAGE (Fig 5C), and Western blotting showed that only TsSPc with 25.18 kDa in ES antigens was identified by anti-rTsSPc serum (Fig 5D). The results demonstrated that TsSPc is expressed in all developmental stages of the nematode, and TsSPc with 25.18 kDa was a secretory protein, which is consistent with that was cloned and expressed in this study.

### Expression and localization of natural TsSPc by IIFT

**Fig 6:**
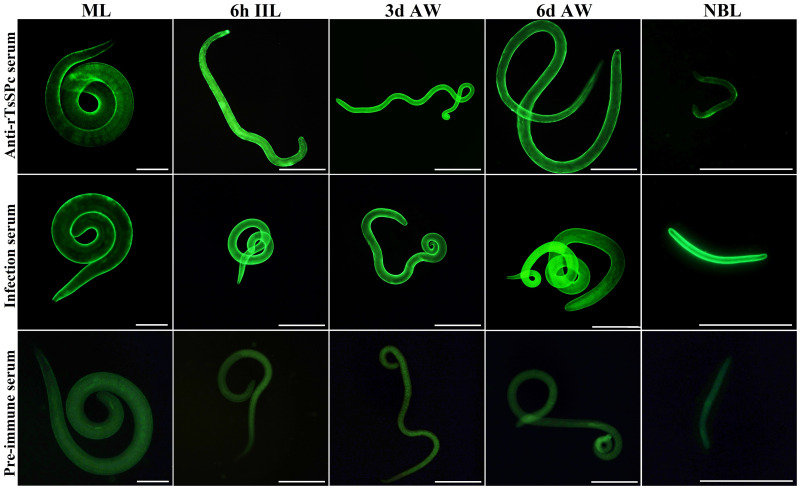
Expression of TsSPc at epicuticle of various *T*. *spiralis* stages by IIFT. Whole worms were probed with anti-rTsSPc serum, and immunofluorescence was detected at the epicuticle of ML, IIL, 3 d AW, 6 d AW and NBL. However, pre-immune normal murine serum did not recognize any worm components of the parasitic nematode. Scale bars:100 μm.

**Fig 7:**
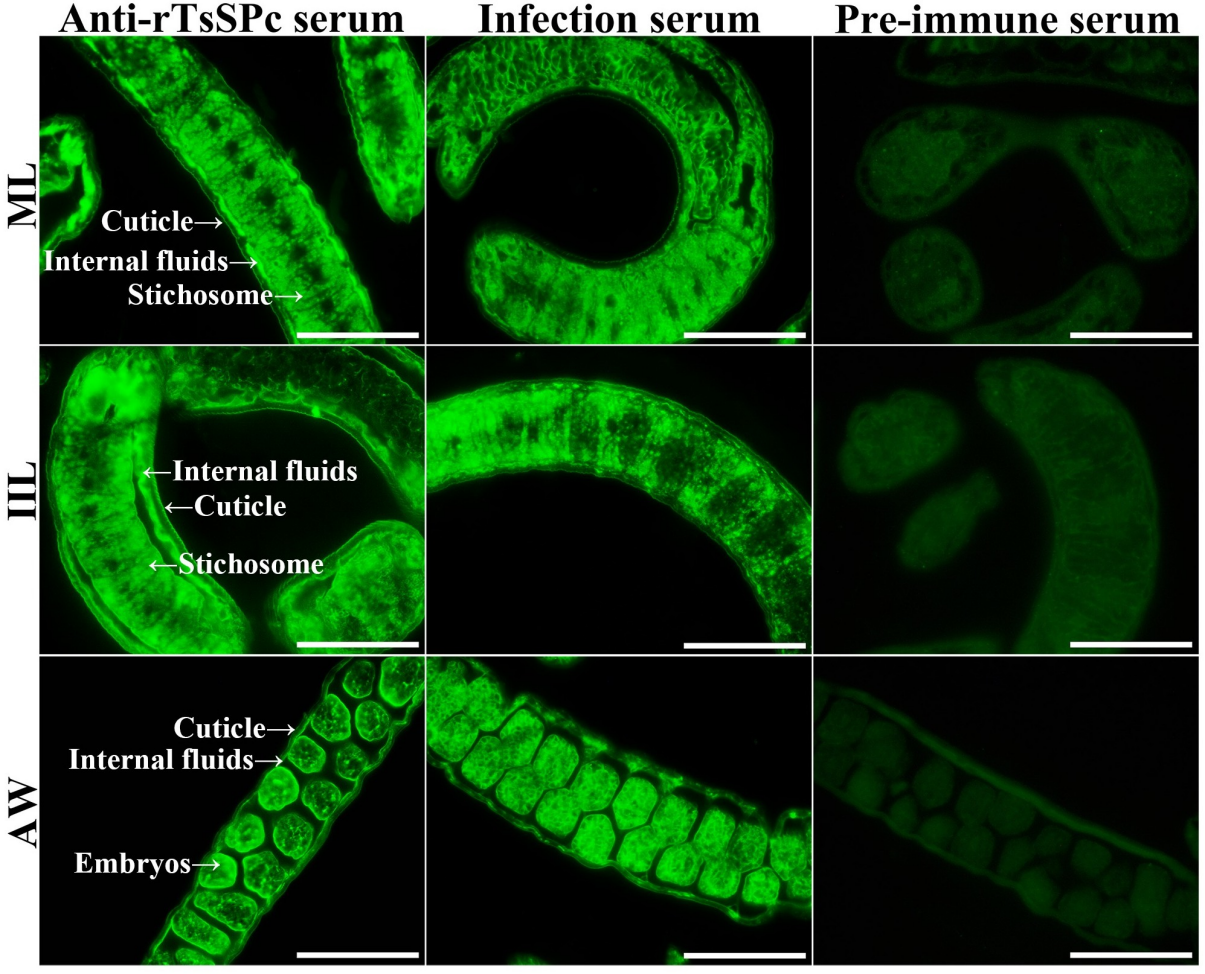
Immunolocalization of TsSPc in different *T*. *spiralis* phases. Fluorescence staining was observed at the cuticle and stichosome of ML, IIL, and embryos of the adult females by using anti-rTsSPc serum. No immunostaining in worm cross-sections was observed by using pre-immune normal serum as a negative control. Scale bars: 40 μm.

The results of IIFT with intact worms showed that green fluorescence was observed on the epidermis of ML, 6h IIL, 3d AW, 6d AW and NBL using anti-rTsSPc serum and infection serum (Fig 6). The IIFT with worm sections revealed that immunofluorescence is mainly localized in the cuticle, stichosome and embryos of the female worms (Fig 7).

### Enzyme activity of rTsSPc

**Fig 8:**
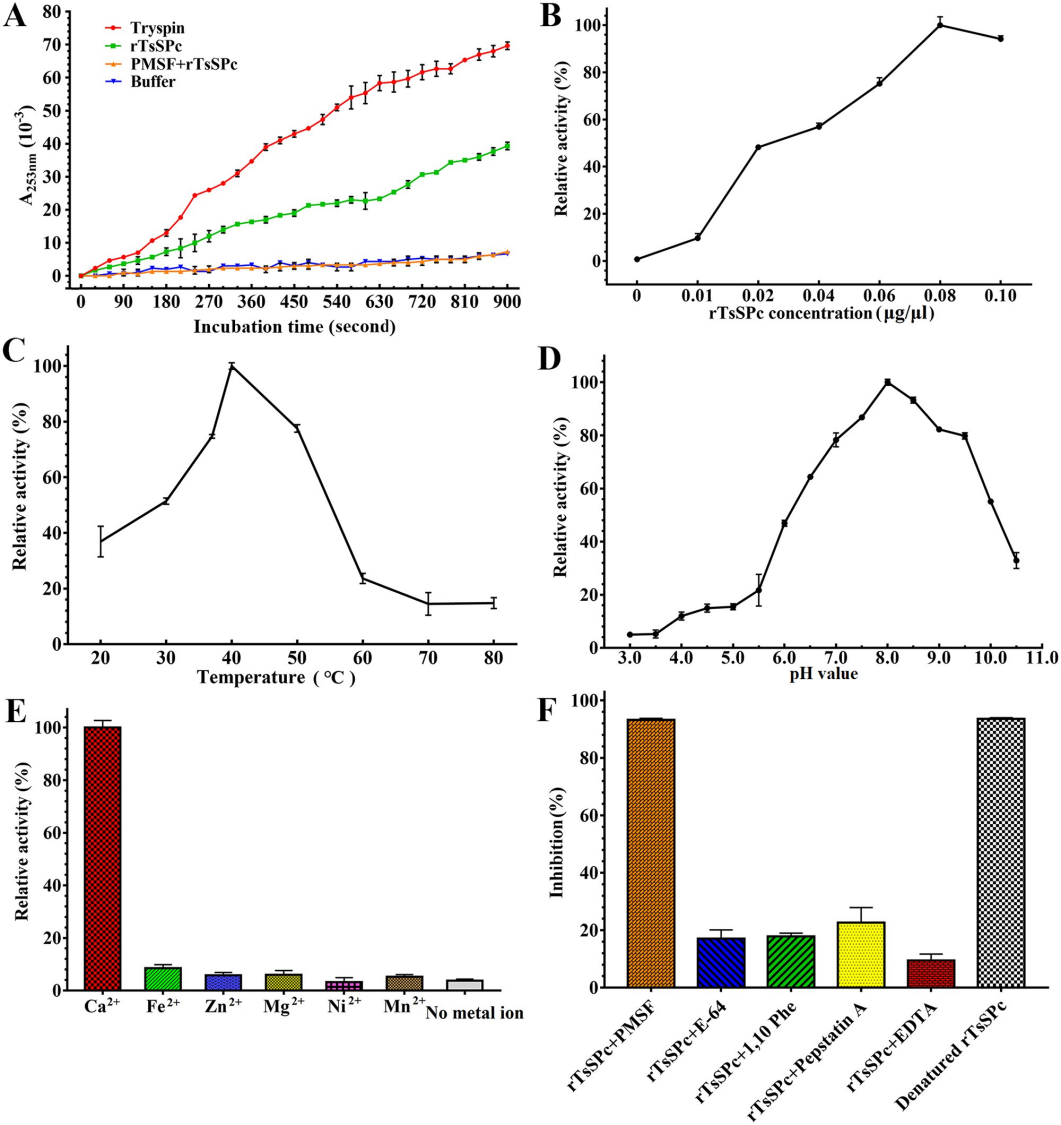
Enzyme activity assay of rTsSPc using BAEE as substrate. rTsSPc was first refolded using dialysis and pre-incubated in different pH (3.0–10.5) buffer solutions and different temperatures (20–80 °C) for 15 min, and its absorbance at 253 nm was measured using a spectrophotometer. **A**: activity of rTsSPc hydrolyzing BAEE increased with prolongation of incubation time. **B**: rTsSPc enzyme activity was detected at different rTsSPc concentrations, and the optimal reaction concentration was 0.08 μg/μl. **C**: rTsSPc activity at different temperatures with the optimal temperature being 40 °C. **D**: The optimum pH for rTsSPc catalyzing BAEE is 8.0. **E**: The effect of different metal ions on the activity of rTsSPc, Ca^2+^ has evident enhancing role on rTsSPc activity. **F**: inhibitory effect of different inhibitors on rTsSPc activity.

The rTsSPc enzyme activity was detected using the substrate BAEE and the activity was increased with prolongation of incubation time (Fig 8A). The rTsSPc enzyme activity was also increased with the increase of rTsSPc concentration. When the rTsSPc concentration was 0.08 μg/μl, the activity tended to be stabilized (Fig 8B), with an optimal temperature of 40 °C (Fig 8C) and an optimal pH of 8.0 (Fig 8D). Ca^2+^ is necessary for rTsSPc enzyme activity (Fig 8E), and PMSF obviously inhibited rTsSPc enzyme activity (Fig 8F).

### Analysis of rTsSPc degrading casein

**Fig 9:**
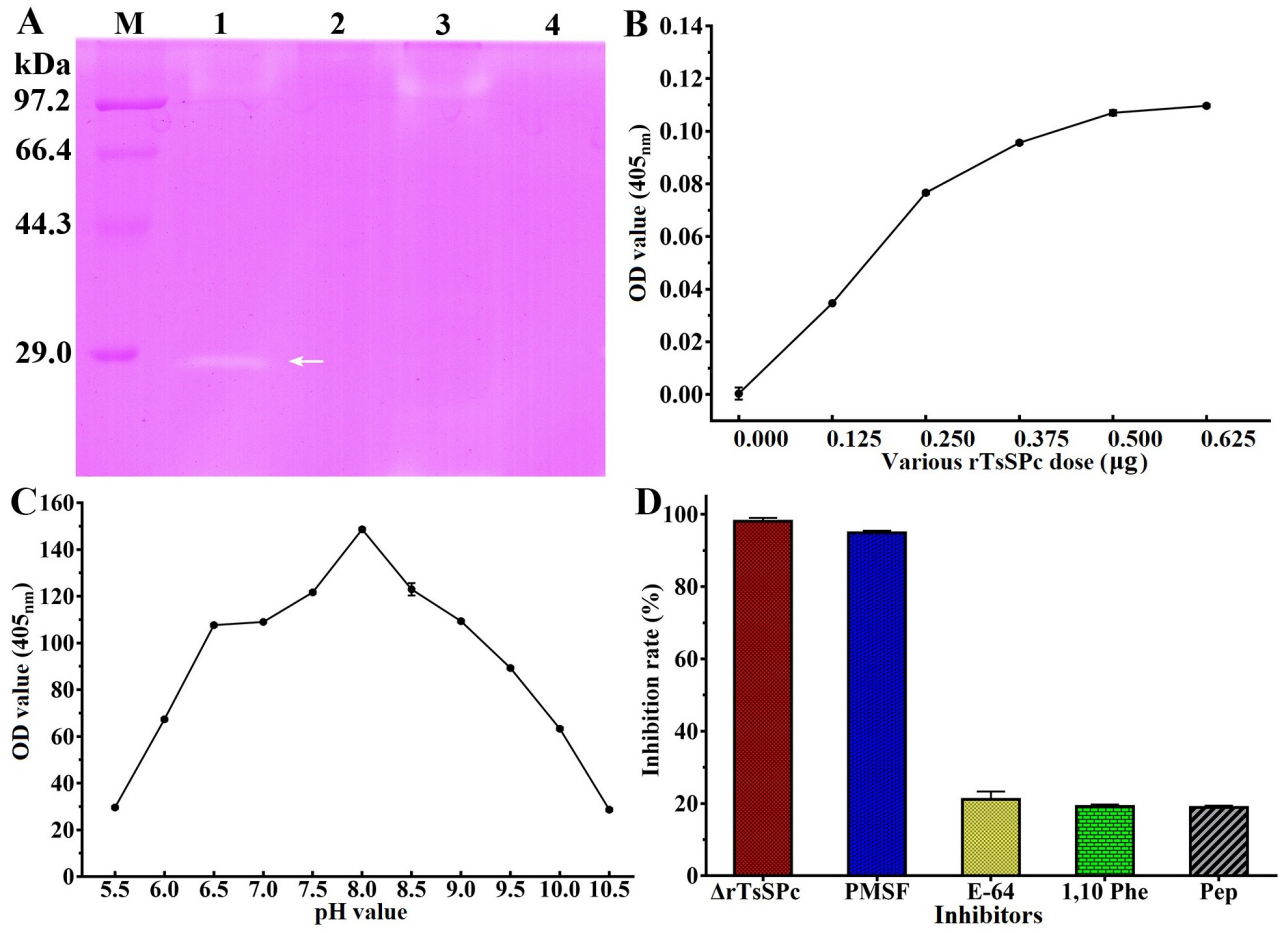
rTsSPc hydrolyzes casein. **A**: Detection of rTsSPc enzyme activity by casein zymography. Lane M: protein mark; lane 1: rTsSPc; lane 2: heat-inactivated rTsSPc; lane 3: rTsSPc+rTsSPI; lane 4: rTsSPc+PMSF. The arrow indicates the 25.18 kDa bands hydrolyzed by rTsSPc. **B**: Different doses of rTsSPc hydrolyze azocasein. **C**: Enzymatic activity of rTsSPc was detected using azocasein at different pH values. **D**: The effect of different inhibitors on rTsSPc protease activity. ΔrTsSPc represents the heat-inactivated rTsSPc at 100 °C for 5 min.

As shown in Fig 9A, there is a clear hydrolysis band at 25.18 kDa, indicating that rTsSPc has the capacity of hydrolyzing casein substrate. But, the denatured rTsSPc did not have the hydrolysis activity. The rTsSPc hydrolyzing activity was inhibited by rTsSPI and PMSF (serine protease inhibitor). The results showed that rTsSPc exhibited the characteristic of serine protease activity. Using azocasein as substrate, different doses of rTsSPc (0, 0.125, 0.250, 0.375, 0.500 or 0.625 μg) were used. The proteolytic activity of rTsSPc was measured within the pH range of 5.5–10.5. The results indicated that the optimal dose of rTsSPc was 0.5 μg (Fig 9B). When pH is 8.0, rTsSPc has the highest hydrolysis activity to azocasein (Fig 9C). After incubation with different enzyme inhibitors, the rTsSPc activity was significantly inhibited by PMSF (Fig 9D). Additionally, the activity of the heat-inactivated rTsSPc was also lost totally.

### Antigenicity and enzymatic activity of MTsSPc

**Fig 10:**
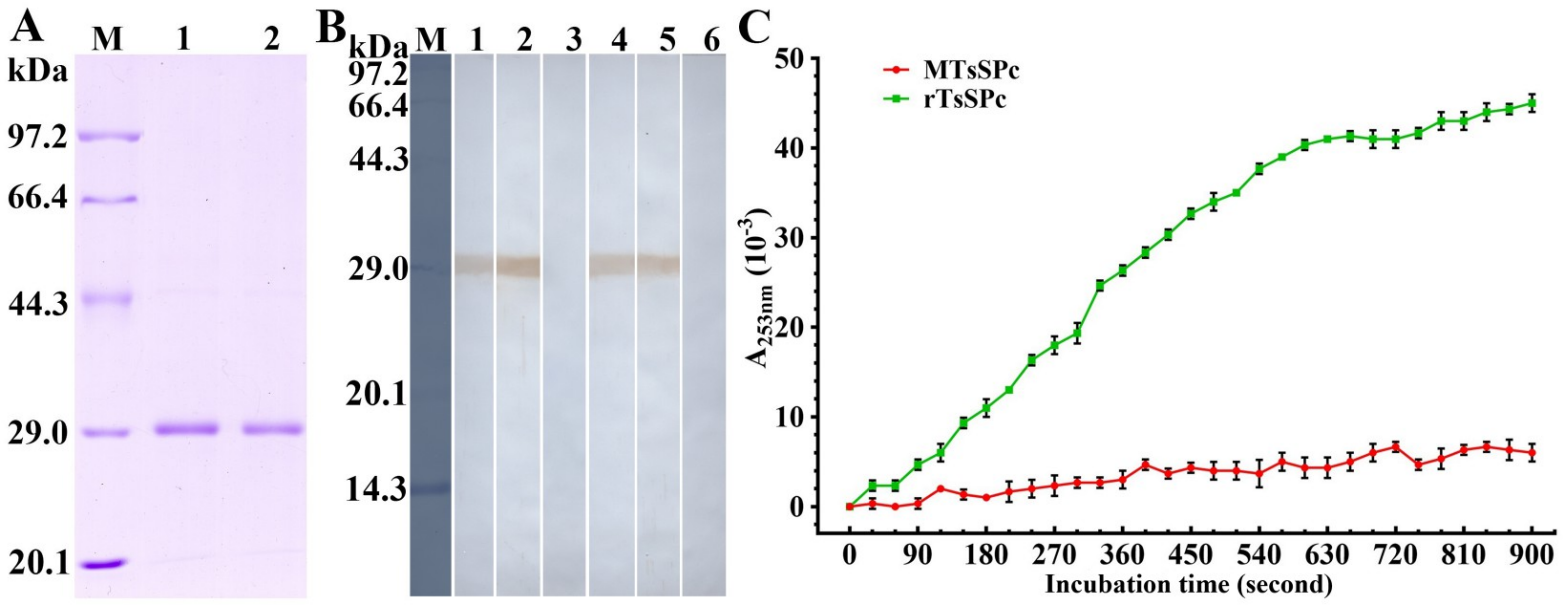
Antigenicity and enzymatic activity of MTsSPc. **A**: SDS-PAGE of MTsSPc. M: protein marker; lane 1: rTsSPc; lane 2: MTsSPc. **B**: Western blot analysis of MTsSPc antigenicity. The rTsSPc was recognized by anti-rTsSPc serum (lane 1), infection serum (lane 2), but not by pre-immune serum (lane 3). The MTsSPc was recognized by anti-rTsSPc serum (lane 4) and murine infection serum (lane 5), but not be recognized by pre-immune serum (lane 6). **C**: Enzymatic activity of MTsSPc was measured using substrate BAEE at the optimal conditions of 37 °C and pH 8.0, and the rTsSPc was used as the positive control.

SDS-PAGE showed that the MW (25.18 kDa) of MTsSPc was the same as the rTsSPc (Fig 10A). Western blot results revealed that the MTsSPc was also recognized by anti-rTsSPc serum and infection serum (Fig 10B), demonstrating that the mutation of TsSPc enzymatic active sites did not affect its antigenic epitopes, and the MTsSPc also had good antigenicity. The enzymatic activity of MTsSPc was determined using substrate BAEE and the result showed that the hydrolytic activity of MTsSPc was completely lost after enzymatic active sites mutation (Fig 10C).

### Cleavage of murine collagen I by rTsSPc

**Fig 11:**
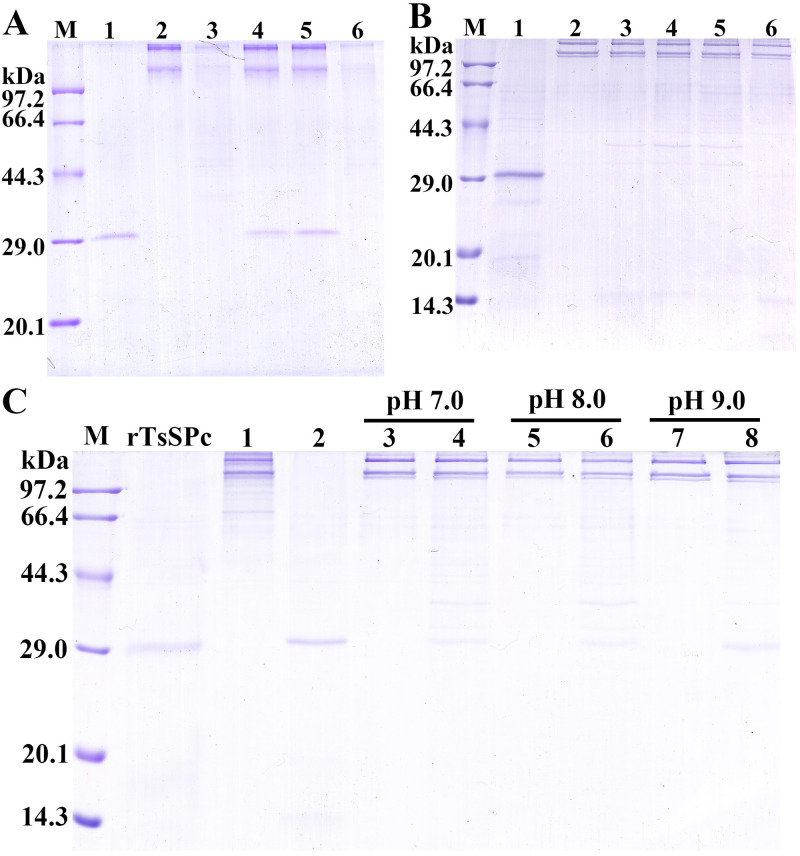
SDS-PAGE analysis of rTsSPc hydrolyzed collagen I. **A**: rTsSPc hydrolyzing collagen I. Lane M: protein marker; lane 1: rTsSPc; lane 2: collagen I; lane 3: collagen I+rTsSPc; lane 4: collagen I+MTsSPc; lane 5: collagen I+PMSF+rTsSPc; lane 6: collagen +trypsin. **B**: The optimal dose of rTsSPc for hydrolyzing collagen I. Lane M: protein marker; lane 2: collagen I; lane 3–6: rTsSPc (0.5, 1.0, 1.5 and 2.0 μg)+collagen I. **C**: Hydrolysis of collagen I using 2.0 μg rTsSPc at different pH values. Lane 1: collagen I; lane 2: rTsSPc; Lanes 3, 5 and 7: collagen I; Lanes 4, 6 and 8: collagen I+rTsSPc.

SDS-PAGE results showed that the collagen I was hydrolyzed by rTsSPc and trypsin, but not by the inhibitor PMSF pre-incubated rTsSPc and MTsSPc (Fig 11A). When various doses of rTsSPc (0.5, 1.0, 1.5 and 2.0 μg) were used, the results showed 2.0 μg rTsSPc had an evident hydrolysis of collagen I, as demonstrated the two bands of collagen I became more thin (Fig 11B, lane 6). The collagen I was hydrolyzed at pH 7.0, 8.0, and 9.0, but the hydrolytic effect of rTsSPc at pH 8.0 was more obvious (Fig 11C, lane 6).

### Binding of rTsSPc and Caco-2 cells determined by Far Western

**Fig 12:**
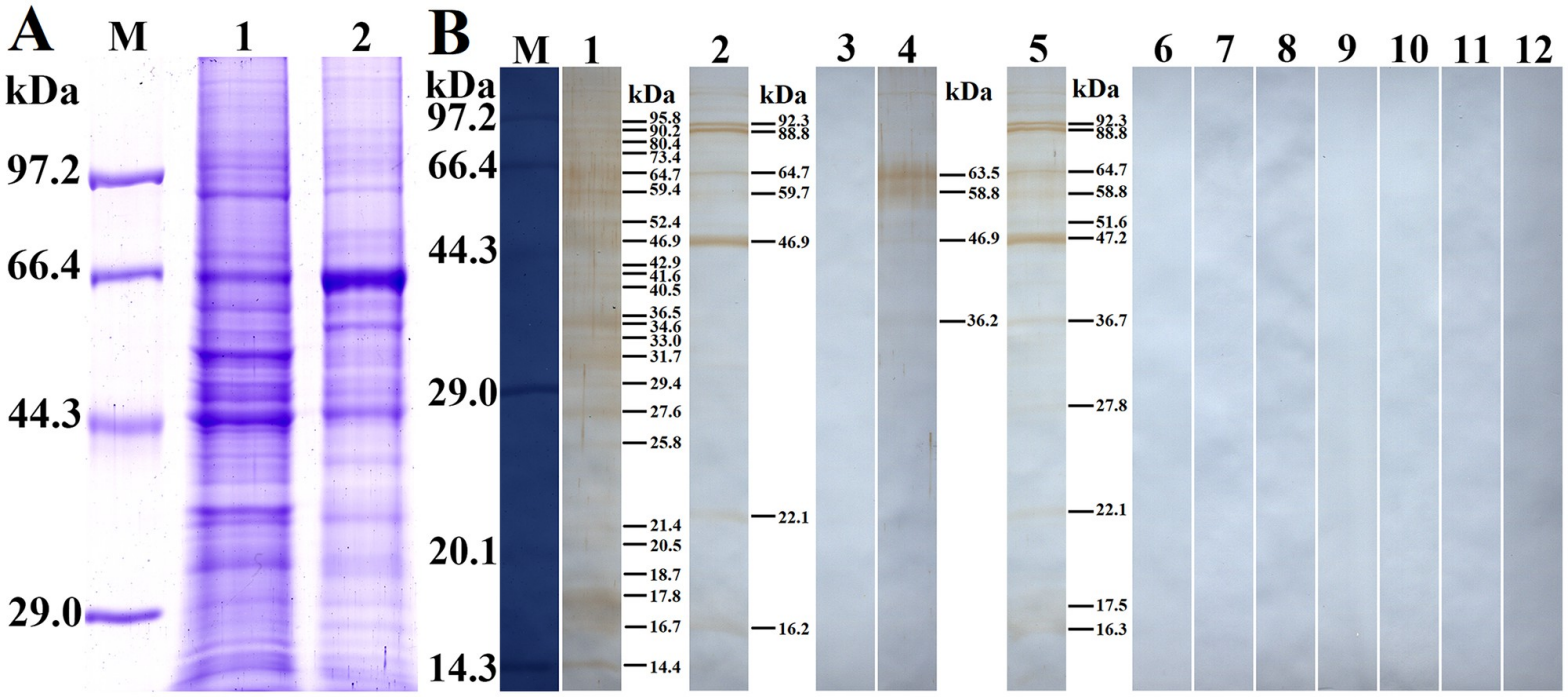
Far-Western analysis of rTsSPc binding to Caco-2 cell proteins. The Caco-2 cell proteins were first analyzed by SDS-PAGE, subsequently the rTsSPc binding with Caco-2 cell protein was detected on Far-Western analysis. **A**: SDS-PAGE analysis of Caco-2 cell proteins. Lane M: protein marker; Lane 1: Caco-2 cell lysates; Lane 2: C2C12 cell lysates as a cell negative control. **B**: Far-Western analysis of rTsSPc binding to Caco-2 cell proteins. Caco-2 cell proteins were first incubated with rTsSPc (Lanes 1–3), IIL ES antigens (Lanes 4–6) or BSA (Lanes 7–9), and then probed by anti-rTsSPc serum (Lanes 1, 4 and 7), infection serum (Lanes 2, 5 and 8), and pre-immune serum (Lanes 3, 6 and 9); The C2C12 protein (Lanes 10–12) was first incubated with rTsSPc, and subsequently probed by anti-rTsSPc serum (Lane 10), infection serum (Lane 11) or pre-immune serum (Lane 12). There was no binding between rTsSPc and C2C12 protein.

Soluble proteins of normal Caco-2 cells were first analyzed by SDS-PAGE, and the results showed that Caco-2 cell lysates had about 24 protein bands of 14.4–95.8 kDa (Fig 12A). Far-Western analysis revealed that after incubation with rTsSPc, all the protein bands of Caco-2 cell lysates were recognized by anti-rTsSPc serum; 7 bands (16.2–92.3 kDa) were recognized by infection serum, but no protein bands were recognized by pre-immune serum (Fig 12B). After Caco-2 cell proteins were incubated with the IIL ES antigens, 4 bands (63.5, 58.8, 46.9 and 36.2 kDa) were identified by anti-rTsSPc serum, whereas 11 bands of 16.3–92.3 kDa were recognized by infection serum, but pre-immune serum did not recognize any bands of Caco-2 cell proteins. The results demonstrated that there is a specific binding between rTsSPc and Caco-2 cell proteins.

### Binding of rTsSPc with Caco-2 and cellular localization by IIFT

**Fig 13:**
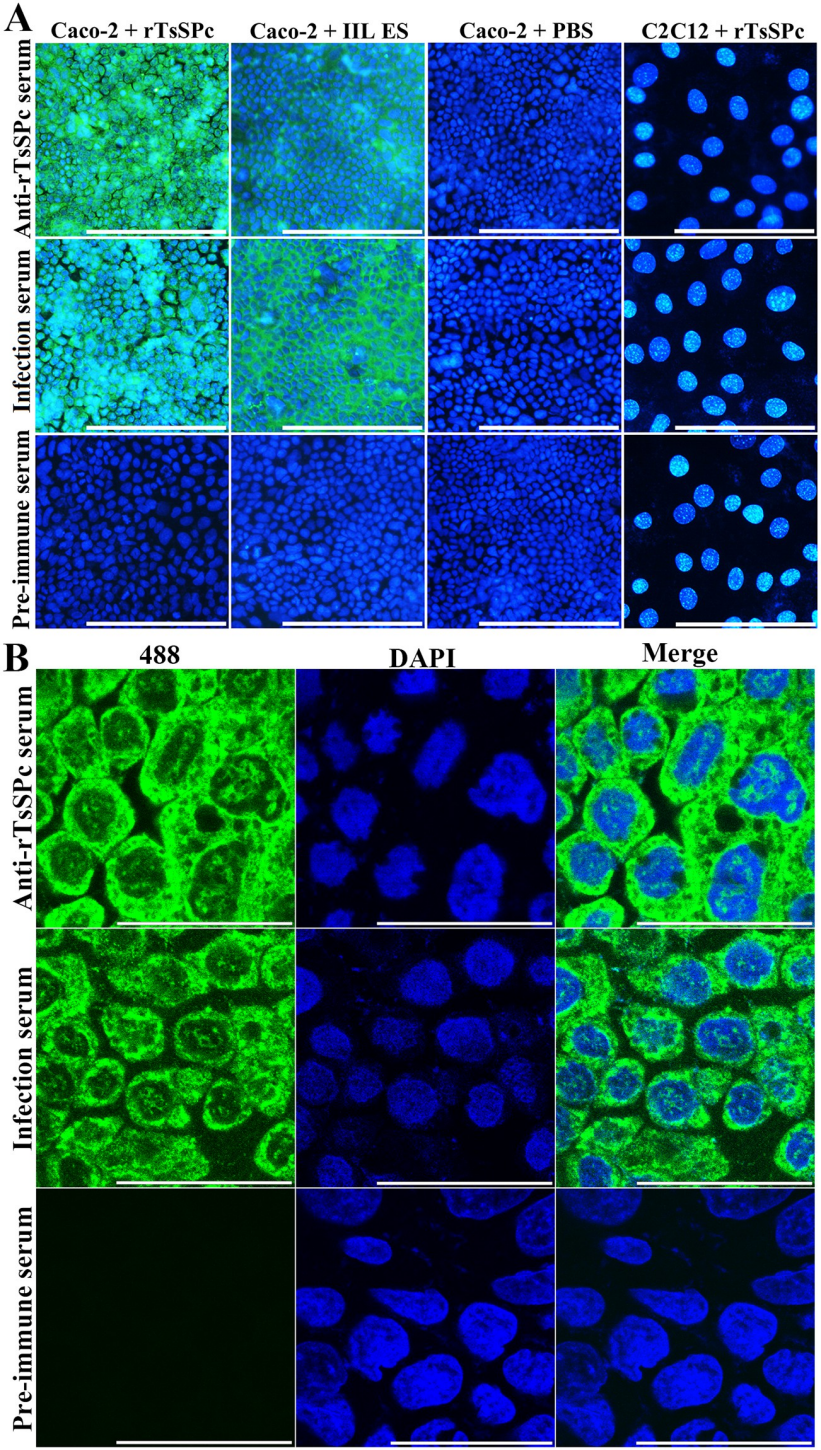
Binding of rTsSPc and Caco-2 cells by IIFT and confocal microscopy. **A**: IIFT analysis of binding between rTsSPc and Caco-2 cells (200×). Caco-2 cells were first incubated with rTsSPc, IIL ES antigens or PBS for 2 h at 37 °C. C2C12 cells were also incubated with rTsSPc for 2 h at 37 °C. After washes, the cells were probed by anti-rTsSPc serum, infection serum or pre-immune serum, subsequently colored with goat anti-mouse IgG-488 conjugate. 4’,6-diamidino-2-phenylindole (DAPI) dyed cell nuclei in blue. Scale bars: 200 μm. **B**: Cellular localization of rTsSPc binding to Caco-2 by confocal microscopy (1000×). The binding sites were localized in cell membrane and cytoplasm Abbreviations: 488: Alexa Fluor 488; DAPI, 4’,6-diamidino-2-phenylindole. Scale bars: 40 μm.

After the Caco-2 cells were incubated with rTsSPc or IIL ES antigens, immune fluorescence was observed on the surface of Caco-2 cells probed by anti-rTsSPc serum or infection serum, but not in the cells probed by pre-immune serum (Fig 13A). However, no fluorescent staining on C2C12 cells incubated with rTsSPc was detected by either anti-rTsSPc serum or infection serum. When positive immune-staining Caco-2 cells were examined by confocal microscopy, the staining was located on Caco-2 cell membrane and cytoplasm, indicating that rTsSPc could specifically bind to the Caco-2 cells, and the binding sites were localized in cell membrane and cytoplasm (Fig 13B).

### Binding of rTsSPc with enteral epithelium

**Fig 14:**
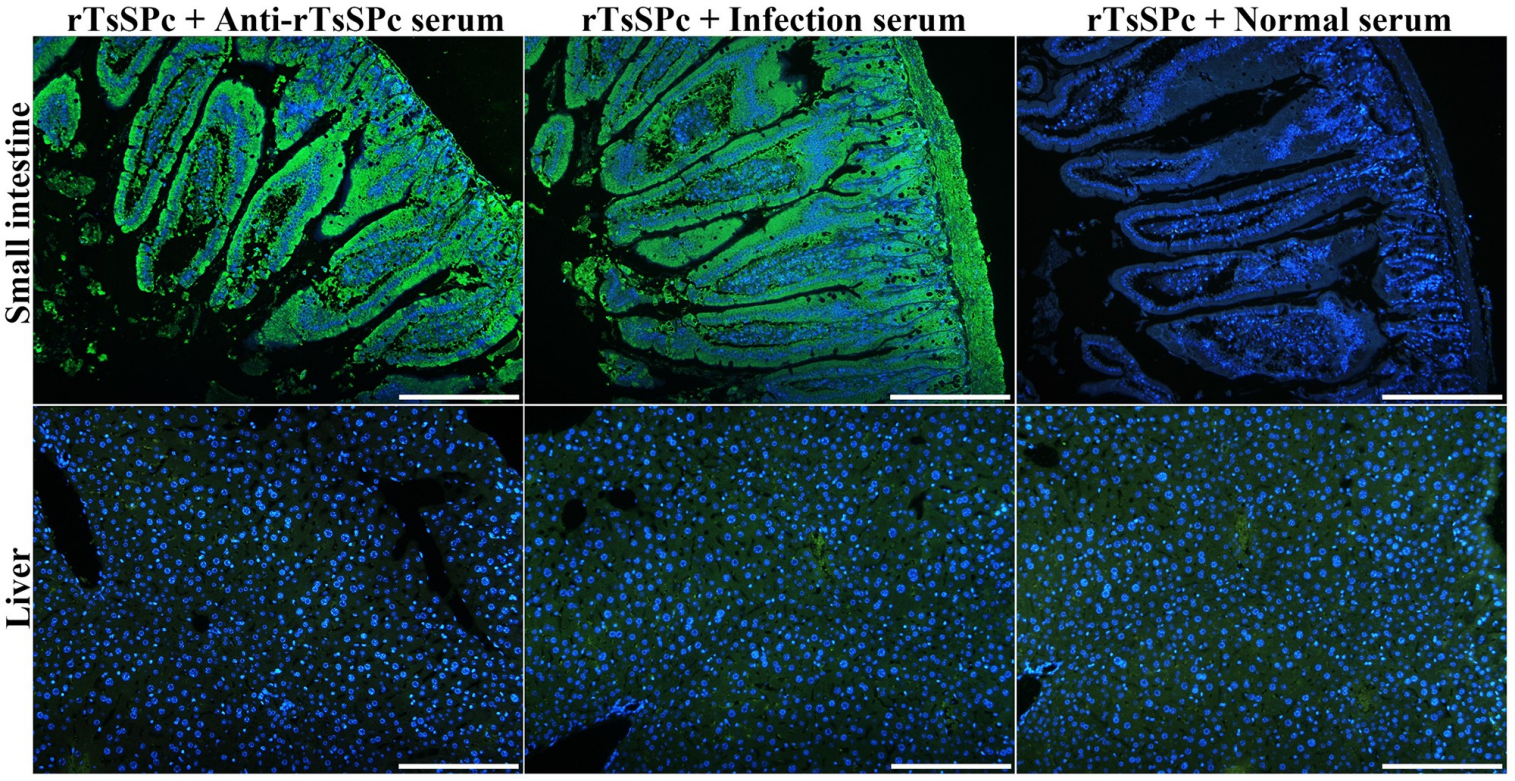
Binding of rTsSPc with normal mouse intestinal epithelium. Intestinal tissue cross sections of normal mice were incubated the rTsSPc, and then probed by anti-rTsSPc serum and infected serum. Intestinal epithelial cell nuclei were stained blue color by DAPI. Scale bars: 200 μm.

The IIFT results showed that after incubation with rTsSPc, green immune fluorescence on normal mouse enteral epithelium was detected by anti-rTsSPc serum and infection serum, but no immunostaining was observed by pre-immune serum (Fig 14). Moreover normal mouse liver cross sections incubated with rTsSPc were not recognized by anti-rTsSPc serum and infection serum. The results indicated that rTsSPc specifically bind to enteral epithelium, and TsSPc might be involved in larval invasion of host intestinal mucosa.

### Reduction of TsSPc expression after silencing TsSPc gene

**Fig 15:**
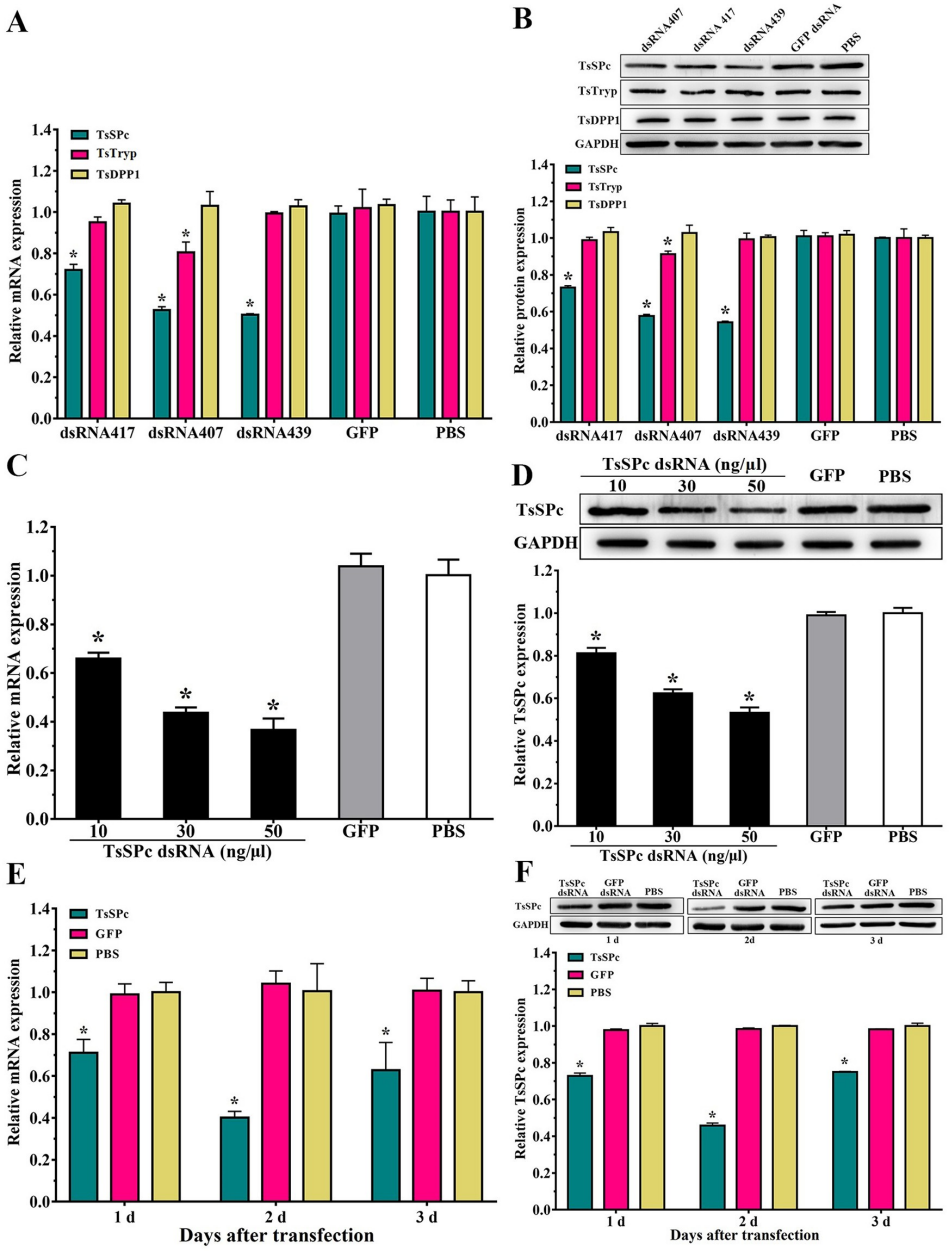
Silencing the TsSPc gene reduces the expression of TsSPc mRNA and protein in *T*. *spiralis*. **A**: Transcription levels of TsSPc, TsTryp and TsDPP1 in ML transfected with three kinks of dsRNA (dsRNA417, dsRNA407 and dsRNA439). **B**: Expression levels of TsSPc, TsTryp and TsDPP1 in ML transfected with three kinds of dsRNA. **C**: Transcription levels of TsSPc genes in ML transfected with different doses of dsRNA. **D**: Expression levels of TsSPc in ML transfected with different doses of dsRNA. **E**: Transcription levels of TsSPc in ML treated with 50 ng/μl dsRNA at 1–3 days after transfection. **F**:Expression levels of TsSPc in ML treated with 50 ng/μl dsRNA at 1–3 days after transfection. *P < 0.05 relative to PBS group.

After *T*. *spiralis* ML transfected with 50 ng/μl dsRNA were cultured for 3 d, larval survival of TsSPc, GFP and PBS group were 80.80, 81.03 and 80.47%, respectively (*F* = 0.363, *P* > 0.05), indicating that electroporation had no significant effect on larval survival. Compared to the GFP and PBS group, the transcriptional and protein expression levels of TsSPc in ML treated with 50 ng/μl dsRNA439 were 0.502 and 0.542 fold (*F* = 103.561, *F* = 609.562, *P <* 0.0001). In addition, transcription and expression of TsTryp and TsDPP1 in ML treated with dsRNA439 were 0.993, 0.994 fold, and 1.03, 1.01 fold, respectively (*F* = 0.14, *P* > 0.05; *F* = 0.162, *P* > 0.05; *F* = 0.375, *P* > 0.05; *F* = 0.893, *P* > 0.05) (Fig 15A and 15B), indicating that the dsRNA439 had no obvious effects on expression of TsTryp and TsDPP1, and dsRNA439 was TsSPc-specific and had good inhibitory effect. The dsRNA439 would be used in the following test. The mRNA and protein expression levels of TsSPc in ML treated with 50 ng/μl dsRNA were 0.366 and 0.532 fold of the PBS group (*F* = 140.191, *F* = 428.373, *P <* 0.0001) (Fig 15C and 15D). After 2 days of transfection with 50 ng/μl dsRNA, the expression levels of TsSPc mRNA and protein were 0.401and 0.458 fold of the PBS group (*F* = 3849.214, *F* = 53.755, *P <* 0.0001) (Fig 15E and 15F), whereas expression level of TsSPc in larvae treated with dsRNA GFP was not evidently affected. Therefore, 50 ng/μl dsRNA 439 was used to transfect the ML, and the culture time following transfection was 2 days to silence the TsSPc gene.

### rTsSPc promoted larval invasion and anti-rTsSPc serum inhibited the invasion

**Fig 16:**
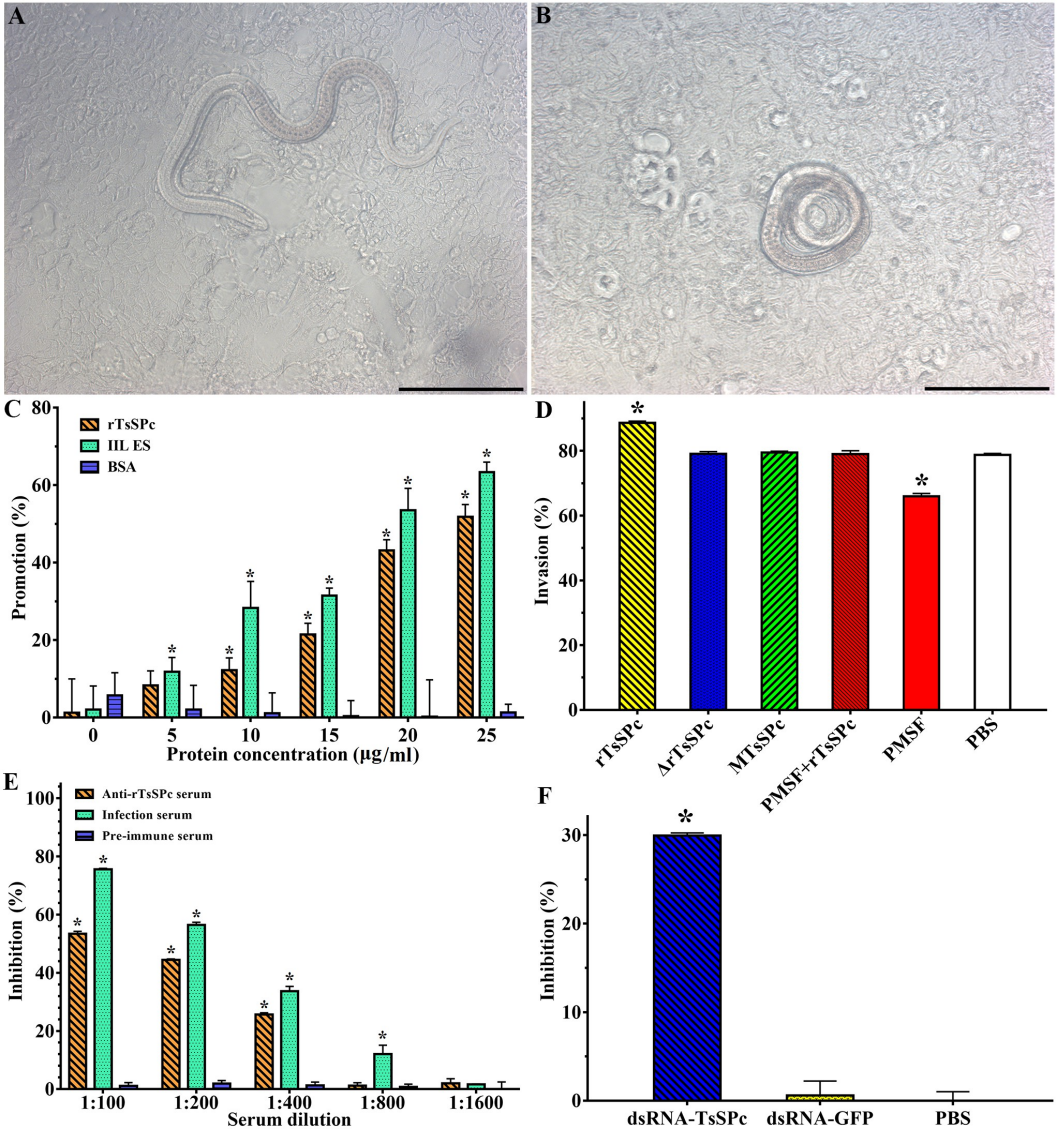
rTsSPc promoted larval invasion and anti-rTsSPc serum and dsRNA inhibited the invasion. The ML was first activated to IIL larvae using 5% swine bile at 37 °C for 2 h, and then added to the Caco-2 cell monolayer. After co-cultivation for 2 h, larval invasion was observed under a microscopy. **A**: invaded larvae in the monolayer were active, and the cell monolayer integrity was disrupted. **B**: non-invaded larvae were spirally coiled on the surface of the cell monolayer. **C**: facilitation of rTsSPc on larval invasion. **D**: heat-inactivated rTsSPc, MTsSPc and PMSF-treated rTsSPc had no any facilitation on the invasion. **E**: inhibition of anti-rTsSPc antibodies on larval invasion. **F**: TsSPc-specific dsRNA suppressed larval invasion. The data were normalized to the PBS group and represented the mean ± SD of three independent tests. Δ rTsSPc represents the heat-inactivated rTsSPc. *P < 0.01 compared to the BSA, PBS or pre-immune serum group. Scale bars: 200 μm.

After the IIL were added to the Caco-2 cell monolayer, the larvae invaded into the monolayer and migrated (Fig 16A and 16B). When the IIL were incubated with various doses of rTsSPc (0, 5, 10, 15, 20 and 25 μg/ml) for 2 h, compared to the PBS group, the facilitation of rTsSPc on larval invasion were 8.32, 12.25, 21.44, 43.16, and 51.82%, respectively (*χ*^*2*^_5_ = 8.333, *P* < 0.01; *χ*^*2*^_10_ = 12.766, *χ*^*2*^_15_ = 23.464, *χ*^*2*^_20_ = 54.777, *χ*^*2*^_25_ = 70.27, *P <* 0.0001). The facilitation showed a dose-dependent of rTsSPc (*r* = 0.969, *P* <0.01), and the BSA had no any facilitation on the invasion (Fig 16C). However, the heat-inactivated rTsSPc, MTsSPc and PMSF-treated rTsSPc had no any facilitation on larval invasion, and the PMSF also abrogated the rTsSPc facilitation on the invasion (Fig 16D), suggested that the rTsSPc facilitation on larval invasion might be related with rTsSPc enzymatic activity. When the culture medium was supplemented with 1:100–1:400 dilutions of anti-rTsSPc serum, its inhibition on larval invasion was 53.56, 44.39 and 25.72%, respectively (*χ*^*2*^_1: 100_ = 70.270, *χ*^*2*^_1: 200_ = 56.410, *χ*^*2*^_1: 400_ = 29.885, *P <* 0.0001). The inhibitory effect of anti-rTsSPc antibody was antibody dose-dependent (*r* = 0.967, *P* < 0.01), and exhibited a decreasing trend with increasing of serum dilution (*F* = 1797.671, *P <* 0.0001) (Fig 16E). In addition, pre-immune serum did not have any inhibitory effects on the larval invasion. After transfection with TsSPc-dsRNA, the invasion of interfered larvae were 50.33%. Compared to the PBS group, the invasion of interfered larvae was reduced by 29.95% (*F* = 699.383, *P <* 0.0001) (Fig 16F). The results indicated that TsSPc plays an essential role during *T*. *spiralis* invasion of host gut epithelium.

## Discussion

Serine proteases (proteinases) are the important members of the proteolytic enzyme superfamily and are widely distributed in organisms. Serine proteases have two main structural folds, the trypsin-like domain and the subtilisin-like domain. Most trypsin-like domains play an important role in assisting parasite invasion, migration, digestion, molting and hydrolysis of host components [64]. Serine proteases of parasites are also involved in reproduction, coagulation, and immune escape. Previous studies have shown that serine proteases from *Trichuris muris* disrupted the integrity of intestinal epithelia due to the hydrolysis of the host intestinal mucus barrier [65]. Specific antibodies against *T*. *spiralis* serine protease 1.1 and 1.2 (TsSP1.1 and TsSP1.2) partially suppressed larval penetration of the IECs, and the rTsSP1.2 triggered an evident immune protection in vaccinated mice [43,66]. Vaccination of mice with rTs31 containing a domain of trypsin-like serine protease induced an obvious antibody response and protection, as demonstrated by a 53.50% ML reduction in challenged mice [62]. RNAi silencing of TsSP1.2 impeded larval invasion of IEC and development in host [67]. These results suggested that serine proteases participated in the larval invasion of intestinal mucosa. However, vaccination of mice with any single serine protease only produced a partial immune protection against larval challenge [17,20]. Therefore, it is necessary to identify and characterize other novel kinds of *T*. *spiralis* serine proteases and evaluate their action in larval invasion of IECs.

In this study, a novel serine proteinase from *T*. *spiralis* (TsSPc) was cloned into pQE-80L plasmid and expressed in *E*. *coli* expression system. Bioinformatics analysis revealed that TsSPc has a signal peptide and a functional trypsin like serine protease domain, and its active site contains the classic catalytic triad. After purification, rTsSPc could be recognized by infection serum, and used to produce anti-rTsSPc antibodies. Immunization of mice with rTsSPc triggered significantly anti-rTsSPc IgG responses, and the titer of serum anti rTsSPc IgG reached 1:10^5^, demonstrating that rTsSPc had good immunogenicity. Moreover, the rTsSPc expressed in a prokaryotic system may have some differences in antigenic epitopes from the natural TsSPc, the rTsSPc was possibly processed through post-translational modifications, alternative splicing or folding, which are possible important for the enzymatic activity and biological functions of the rTsSPc [32,34,68]. The catalytic activity of serine proteases is crucial for the parasite invasion of host tissues. Previous studies showed that BAEE, casein, and collagen I could be hydrolyzed by serine proteases [69]. Therefore, in this study, casein, BAEE and collagen I were used as substrates to evaluate the enzyme activity of rTsSPc. The results of casein gel zymography showed that rTsSPc could hydrolyze casein substrate, and a clearly hydrolytic band at 25.18 kDa was consistent with the corresponding MW of rTsSPc. The results of rTsSPc enzyme activity assay revealed that rTsSPc had the enzyme activity of natural serine protease for hydrolyzing the substrate BAEE at 40 °C and pH 8.0, and the enzyme activity was higher in weakly alkaline environments. Additional, rTsSPc hydrolyzed collagen I was dose-dependent at pH 8.0. The results were consistent with the optimal pH values of most serine proteases from other parasites previously reported [70]. Serine proteases in *Fasciola hepatica* miracidia hydrolyzed azocasein with optimum activity at pH 8.0. The optimum pH effect on serine proteases activities was found at alkaline pH [71]. Activity of *Eimeria tenella* serine proteases to degrade casein substrate was observed at pH 8.2 [72]. The activity of a recombinant serine proteinase from *T*. *spiralis* muscle larvae was also detected at the optimal pH 8.0 [73]. Ca^2+^ significantly enhanced the enzyme activity of rTsSPc, indicating that TsSPc activity was Ca^2+^ dependent, while other metal ions had no obvious impact on rTsSPc activity. MTsSPc and rTsSPc had the same molecular weight, and recognized by rTsSPc immune serum, but the catalytic activity of MTsSPc was completely lost. The results further verified the catalytic properties of TsSPc.

qPCR showed that expression of TsSPc mRNA was observed at different *T*. *spiralis* stages (ML, IIL, 3d and 6d AW, and NBL), indicating that the TsSPc gene was transcribed throughout all developmental stages of the nematode. Western blotting results showed that rTsSPc was recognized by anti rTsSPc serum and mouse infection serum. Two clear bands (28.33 and 25.18 kDa) in crude antigens of various worm stages were recognized by anti-rTsSPc serum, suggesting that one might be precursor (e.g., trypsinogen, 28.33 kDa) and another was the activated state (mature serine protease, 25.18 kDa) of TsSPc with enzyme activity after activation [74]. Western blot results also showed that TsSPc was a secreted protein. The expression level of TsSPc at 3 d AW stage was significantly higher than that of other stages (ML, IIL, 6d AW, and NBL). The IIFT results showed that natural TsSPc was also expressed at various stages and principally localized in epicuticle, stichosome and intrauterine embryos of this nematode. The stichosome is a secretory organ of the nematode, further indicating that TsSPc was a secretory and surface protein [75]. The results suggested that TsSPc might be exposed to the host gut epithelium at early stage of *T*. *spiralis* infection, and participated in destruction of gut epithelium integrity and larval invasion of intestinal mucosa [6,76,77].

Far Western blot analysis was usually used to ascertain protein-protein interaction from the protein mixtures and has been successfully used to verify the binding of *T*. *spiralis* proteins to IECs [10,12]. In this study, the results of Far-Western blot and IIFT indicated that there was a specific binding between rTsSPc and Caco-2 cells, confocal microscopy showed that the binding sites of rTsSPc and Caco-2 cells were mainly localized in cellular membrane and cytoplasm. The IIFT results also showed that rTsSPc could bind to intestinal epithelium of normal mice. Previous studies have shown that when the IIL were co-incubated with IECs, the IIL secreted some new proteases which entered into the IEC [39,78]. The proteases might be the *Trichinella* invasion-related molecules. Nonetheless, what kind of IEC proteins bound with rTsSPc was not clear, it is necessary to be investigated by co-immunoprecipitation and mass spectrometry in further study.

The RNAi induced transcriptional gene silencing, thereby reduced the gene expression of parasites. RNAi is a good way to investigate the gene function of parasitic nematodes [79]. After silencing the *Brugia malayi* cysteine protease, female embryos were found to have dysplasia and abnormal structure [80]. When the cathepsin of *Ceratophyllum* is silenced by RNAi, the larval molting is significantly inhibited, indicating that cathepsin plays an important role in larval molting [81]. RNAi silencing of *Fasciola hepatica* cysteine protease genes (FhCatB1 and FhCatL1) significantly reduced the juvenile invasion of intestinal mucosa [82]. Silencing of some *T*. *spiralis* genes resulted in an evident inhibition on larval invasion of IECs, and worm development and reproduction were also hindered [83–85]. The dsRNA or siRNA is usually used to silence parasite genes, and the methods of introducing them into parasites have immersion, electroporation and microinjection. In this study, we designed the specific long strand dsRNA of TsSPc, and introduced the dsRNA into the ML by electroporation. When 50 ng/μl TsSPc-dsRNA was introduced into ML and the worms were cultured for 2 days, silencing of TsSPc gene was obvious. Our results showed that specific dsRNA significantly reduced the TsSPc expression and impeded the larval penetration of the cell monolayer, suggesting that TsSPc exerted a crucial action in the process of larval invasion of gut epithelium.

The results of the *in vitro* larval invasion assay showed that rTsSPc promoted larval invasion of Caco-2 cells, while anti-rTsSPc antibodies inhibited larval invasion. The promotion or inhibition is dose-dependent of rTsSPc and anti-rTsSPc antibodies. Furthermore, the heat-inactivated rTsSPc, MTsSPc and PMSF pre-incubated rTsSPc had no any facilitation on larval invasion, and the PMSF also abrogated the rTsSPc facilitation on the invasion. The results suggested that the rTsSPc facilitation on larval invasion might be related with the binding of rTsSPc to Caco-2 cells and enzymatic activity of rTsSPc [14,16,86]. The inhibition of larval invasion of Caco-2 by anti rTsSPc antibodies may be due to the formation of a cap like immune complex between TsSPc and anti-TsSPc antibodies at the worm anterior, blocked direct contact between worms and gut epithelium, therefore hindered larval invasion [17,48]. The results further indicated that TsSPc participated in *T*. *spiralis* larval invasion of gut epithelium; TsSPc might be a main invasive protein molecule, and it might be considered as candidate vaccine target against *Trichinella* invasion.

*Trichinella spiralis* is a foodborne zoonotic parasitic nematode, and *T*. *spiralis* infection is resulted from ingesting raw or undercooked infected animal meat. The IIL is the first invasive stage in the life cycle of *T*. *spiralis* [21]. Our results showed that rTsSPc mediated the IIL invasion of IECs, whereas anti-rTsSPc antibodies obviously impeded larval invasion. When larval invasion is impeded, the IIL development will be interrupted; consequently *T*. *spiralis* infection will be blocked [19,20]. Therefore, the rTsSPc would be a potential candidate of preventive vaccines against *T*. *spiralis* invasion and infection. Anti-*Trichinella* vaccines provided a prospective strategy for control and elimination of *Trichinella* infection in food animals [35].

However, this study still has some limitations, for examples, rTsSPc bound to what kinds of proteins in IECs has not been identified, the mechanisms of TsSPc promoting larval invasion of IECs have not been elucidated, and the protective immunity elicited by immunization of mice with rTsSPc has not been evaluated. These issues need to be investigated in the further study.

In conclusion, TsSPc is highly expressed in intestinal adult worm phase of *T*. *spiralis* life cycle, mainly located at cuticle, stichosome and intrauterine embryos of this nematode. TsSPc is a surface and secretory protein. The rTsSPc has good immunogenicity and the enzymatic activity of native serine protease, which hydrolyzed the substrate BAEE, casein and collagen I. rTsSPc specifically bound to IECs and the binding sites were mainly localized in cell membrane and cytoplasm. rTsSPc accelerated larval invasion of IEC, while anti-TsSPc antibodies obviously hindered larval invasion, the acceleration and suppression are dose-dependent of rTsSPc protein and anti-TsSPc antibodies. Silencing of the TsSPc gene by TsSPc-specific dsRNA significantly reduced the expression of rTsSPc in the nematode and impaired larval invasion of IECs. The results indicated that TsSPc plays an important role in larval invasion of host gut mucosa, and may be considered as a candidate target molecule for the vaccines against *Trichinella* intrusion.
